# O304 ameliorates hyperglycemia in mice by dually promoting muscle glucose effectiveness and preserving β-cell function

**DOI:** 10.1038/s42003-023-05255-6

**Published:** 2023-08-25

**Authors:** Stefan Norlin, Jan Axelsson, Madelene Ericsson, Helena Edlund

**Affiliations:** 1 Umeå Centre for Molecular Medicine, Umeå University, SE-901 87 Umeå, Sweden; 2 Department of Radiation Sciences, Radiation Physics, Umeå University, SE-901 87 Umeå, Sweden

**Keywords:** Diabetes, Diabetes

## Abstract

Although insulin mediated glucose uptake in skeletal muscle is a major mechanism ensuring glucose disposal in humans, glucose effectiveness, i.e., the ability of glucose itself to stimulate its own uptake independent of insulin, accounts for roughly half of the glucose disposed during an oral glucose tolerance test. Both insulin dependent and insulin independent skeletal muscle glucose uptake are however reduced in individuals with diabetes. We here show that AMPK activator O304 stimulates insulin independent glucose uptake and utilization in skeletal muscle and heart in vivo, while preventing glycogen accumulation. Combined glucose uptake and utilization requires an increased metabolic demand and we show that O304 acts as a mitochondrial uncoupler, i.e., generates a metabolic demand. O304 averts gene expression changes associated with metabolic inflexibility in skeletal muscle and heart of diabetic mice and reverts diabetic cardiomyopathy. In Type 2 diabetes, insulin resistance elicits compensatory insulin hypersecretion, provoking β-cell stress and eventually compensatory failure. In db/db mice O304 preserves β-cell function by preventing decline in insulin secretion, β-cell mass, and pancreatic insulin content. Thus, as a dual AMPK activator and mitochondrial uncoupler O304 mitigates two central defects of T2D; impaired glucose uptake/utilization and β-cell failure, which today lack effective treatment.

## Abstract (web summary)

Small molecule O304, by dually activating AMPK and mitochondrial uncoupling, ameliorates central T2D defects in glucose disposal and glucose use in skeletal muscle and heart, and preserves pancreatic beta-cell function

## Introduction

Skeletal muscle is the major organ accountable for postprandial glucose uptake and thus largely contributes to maintenance of glucose homeostasis under normal conditions^1,2^. In man, roughly half of the glucose disposal during an oral glucose tolerance test is mediated by insulin^3^. The mass action effect of glucose, also known as glucose effectiveness, i.e., the ability of glucose itself to stimulate its own uptake independent of insulin, accounts for the remaining half of glucose disposal^3–8^. Under conditions of insulin resistance, as in type 2 diabetes (T2D), or insulin deficiency, as in type 1 diabetes (T1D), insulin stimulated skeletal muscle uptake is reduced. However, in both T1D and T2D subjects, glucose effectiveness is also reduced, referred to as glucose resistance^9^. No anti-diabetic drug in clinical use mitigates skeletal muscle insulin resistance or enhances glucose effectiveness in diabetic individuals, although both should be considered as valuable strategies for treatment of diabetes.

AMPK activation increases insulin independent glucose uptake in vitro in myotubes^10–12^ and is also implicated in the increased glucose effectiveness observed post-exercise in endurance and strength-trained humans^13,14^. Consequently, pharmacological activation of AMPK has been proposed as an attractive approach to treat T2D. Direct allosteric AMPK activation by compounds that binds the so called AdAM site in AMPK increases glucose uptake in skeletal muscle in an insulin-independent manner but fail to increase tricarboxylic acid (TCA) flux and glucose oxidation, and promote accumulation of glycogen in skeletal muscle and heart^11,12^. Thus, compared to exercise/contraction, AdAM site AMPK activators fail to generate the metabolic demand, i.e., ADP increase, that promotes TCA flux, increased mitochondrial respiration, and glucose oxidation^15^. In contrast, O304 increased glucose uptake, cardiac function, and exercise capacity in young and aged mice, without promoting glycogen accumulation in heart^10,16^, i.e., acting like a bona fide exercise mimetic. O304 also prevented and reverted obesity in diet-induced obese (DIO) mice and induced a switch from carbohydrates to fatty acids as main energy source, indicating that O304 increases energy expenditure by generating a metabolic demand, either by inducing futile cycling and/or mitochondrial uncoupling^10^.

Treatment of T2D also involves strategies aiming at stimulating insulin secretion from already metabolically stressed β-cells, i.e., using various secretagogues, which in many cases has been associated with a “treat-to-failure” scenario with accelerated β-cell stress and eventual loss of β-cell function^17,18^. In contrast, multiple approaches to lower the demand for insulin secretion and thus β-cell stress, show that β-cell dysfunction, at least if not progressed too far, is reversible^19–26^. Thus, induction of β-cell rest and preservation of glucose responsive, insulin secreting β-cells has emerged as an attractive complementary therapeutical approach for treatment of T2D^17,20,21^. Hyperglycemia increases Thioredoxin-interacting protein (TXNIP) levels and elevated TXNIP levels are markers of glucotoxicity effects including reduced skeletal muscle and cardiac glucose uptake, diabetic cardiomyopathy, and impaired β-cell function^27–31^. AMPK negatively regulates TXNIP levels both directly via phosphorylation of TXNIP^28^, and indirectly by phosphorylation inhibition of ChREB, a transcription factor regulating TXNIP expression^32^.

Here we have assessed the in vivo effects of the clinical stage, AMPK activator O304 in two distinct diabetic mouse models with respect to (i) glucose effectiveness and (ii) β-cell function. O304 stimulated glucose uptake and utilization in skeletal muscle in vivo under insulin deficient conditions and mitigated hyperglycemia associated glycogen accumulation in skeletal muscle and heart. We also show that O304 acts as a mitochondrial uncoupler in differentiated myotubes ex vivo, thus generating a metabolic demand promoting glucose utilization. O304 averted gene expression changes associated with metabolic inflexibility in skeletal muscle and heart of diabetic mice and reverted diabetic cardiomyopathy. Moreover, O304 preserved β-cell function in an in vivo insulin resistant context and under in vitro conditions of chronic hyperglycemia. The dual mechanism of action and the dual anti-hyperglycemic properties exerted by O304, i.e., (i) stimulation of glucose effectiveness together with (ii) preservation of β-cell function under hyperglycemic conditions, holds great potential for treatment of diabetes provoked both by insulin resistance and insulin deficiency.

## Results

### O304 prevents development of diabetes and reduces established hyperglycemia in STZ mice

**Fig. 1:**
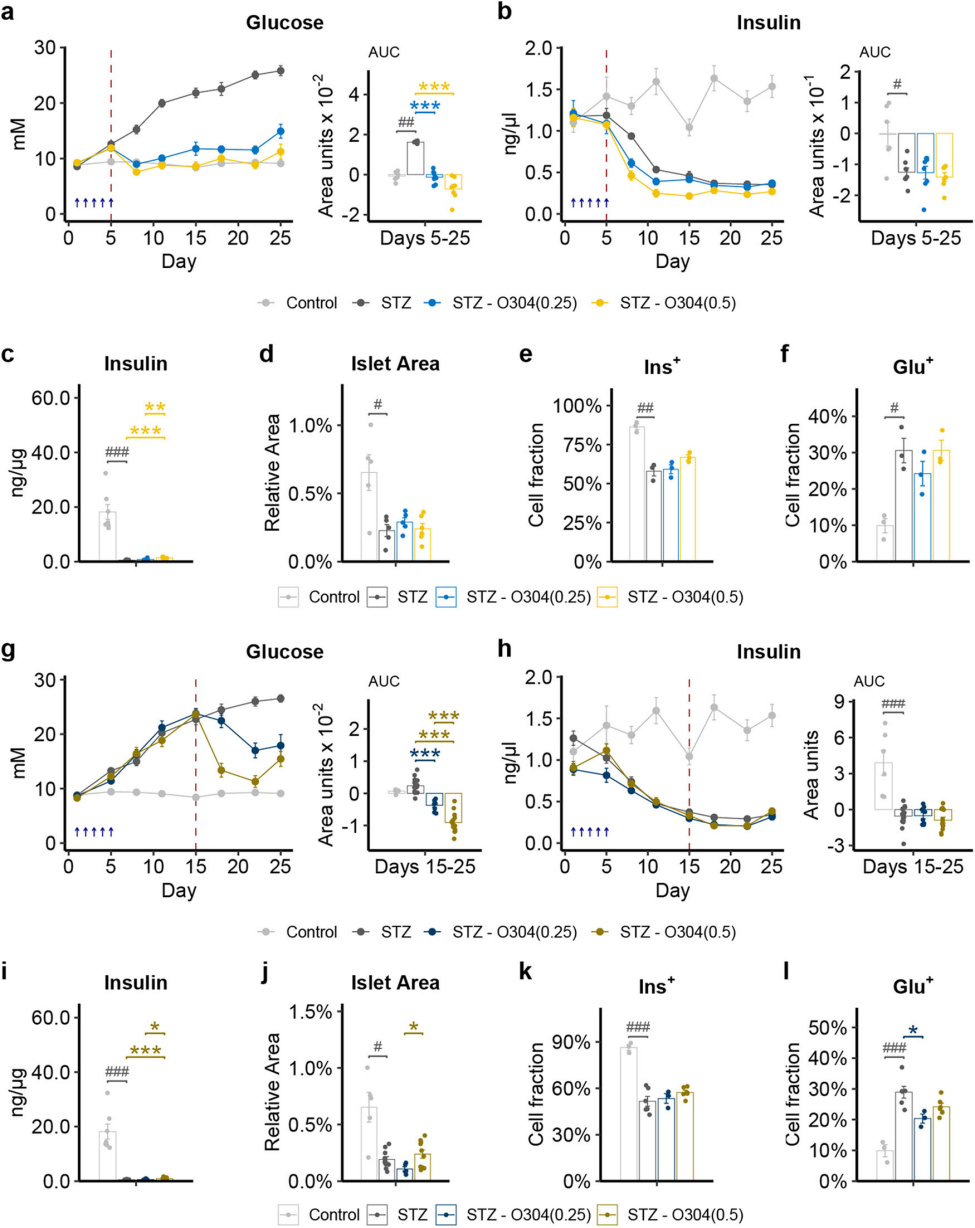
O304 averts hyperglycemia in STZ mice. **a**, **b** Fasted glucose (**a**) and insulin (**b**) levels with area under the curve (AUC) in control (*n* = 7) and STZ mice untreated or treated with 0.25 and 0.5 mg/g O304, respectively, from day 5 (*n* = 6–8/group). **c** Total pancreatic insulin content in control and STZ mice untreated or treated with 0.25 and 0.5 mg/g O304, respectively, from day 5 (*n* = 7–8/group). **d**–**f** Islet cell area (**d**), Insulin positive (Ins^+^) (**e**), and Glucagon positive (Glu^+^) (**f**) cell fraction in control (*n* = 3–5) and STZ mice untreated (*n* = 3–5) or treated with 0.25 (*n* = 3–5) and 0.5 (*n* = 3–6) mg/g O304, respectively, from day 5. **g**, **h** Fasted glucose (**g**) and insulin (**h**) levels with area under the curve (AUC) in control (*n* = 7) and STZ mice untreated or treated with 0.25 and 0.5 mg/g O304, respectively, from day 15 (*n* = 8–16/group). **i** Total pancreatic insulin content in control (*n* = 7) and STZ mice untreated or treated with 0.25 and 0.5 mg/g O304, respectively, from day 15 (*n* = 8–16/group). **j**–**l** Islet cell area (**j**), Ins^+^ (**k**) and Glu^+^ cell fraction (**l**) in control (*n* = 3–5), STZ mice untreated (*n* = 6–10) or treated with 0.25 (*n* = 3–5) and 0.5 (*n* = 6–10) mg/g O304, respectively, from day 15. Please note that control mice in (**a**–**f**) are the same as in corresponding panels in (**g**–**l**). Data are presented as mean ± SEM. Statistical significance between untreated and O304-treated STZ mice was determined by Welch’s ANOVA followed by Games–Howell post hoc test (**a**–**d**, **g**–**j**) or one-way ANOVA followed by Tukey’s post hoc test (**e**, **f**, **k**, **l**) (*P < 0.05, **P < 0.01, ***P < 0.001) and between control mice and STZ mice by Wilcoxon test (**a**–**d,** **g–j**) or by Student’s *t* test (**e**, **f**, **k**, **l**) (^#^*P* < 0.05, ^##^*P* < 0.01, ^###^*P* < 0.001).

To assess whether O304 could enhance glucose effectiveness and thus ameliorate diabetes in an insulin deficient context, diabetes was induced by ablating β-cell function through streptozotocin (STZ) injections in 9–11 weeks (w) old male C57BL/6J × CBA F1 mice. At day 5 of the last STZ injection, 6 h fasted glucose levels were slightly increased and insulin levels were moderately decreased (Fig. 1a, b). STZ mice were next treated with a diet formulated with 0.25 or 0.5 mg/g for O304, O304(0.25) and O304(0.5), respectively, from day 5 or 15. In untreated STZ mice insulin levels continued to decrease with a concomitant increase in glucose levels, resulting in development of overt diabetes (Fig. 1a, b). In contrast, STZ mice treated with O304 from day 5 rapidly normalized glucose levels (Fig. 1a) without increasing plasma insulin levels (Fig. 1b). Thus, normalization of glucose levels in O304-treated STZ mice is insulin independent. Consequently, pancreatic insulin content was 10–20-fold lower in O304-treated mice compared with non-diabetic control mice (Fig. 1c and Supplementary Fig. 1b). Islet cell area and Ins^+^ cell fraction were decreased, and Glu^+^ islet cell fraction increased, to similar extent in untreated and O304-treated STZ mice compared to control mice (Fig. 1d–f and Supplementary Fig. 1a, c, d). O304 rapidly reduced glucose levels without enhancing plasma insulin levels also in STZ mice where the treatment was initiated first at day 15, i.e., when the mice had developed overt diabetes (Fig. 1g and Supplementary Fig. 1e) (Fig. 1h). Pancreatic insulin content was again lower, ~20-fold, in O304-treated mice compared with that of control mice (Fig. 1i and Supplementary Fig. 1g). Similarly, islet cell area and Ins^+^ islet cell fraction was reduced and Glu^+^ islet cell fraction increased in untreated and O304-treated STZ mice compared to that of control mice (Fig. 1j–l and Supplementary Fig. 1f, h, i). Together these findings show that O304 potently and in an insulin independent manner reverts diabetes in an insulin deficient mouse model of diabetes.

### O304 ameliorates hyperglycemia in STZ mice by stimulating muscle glucose uptake

**Fig. 2:**
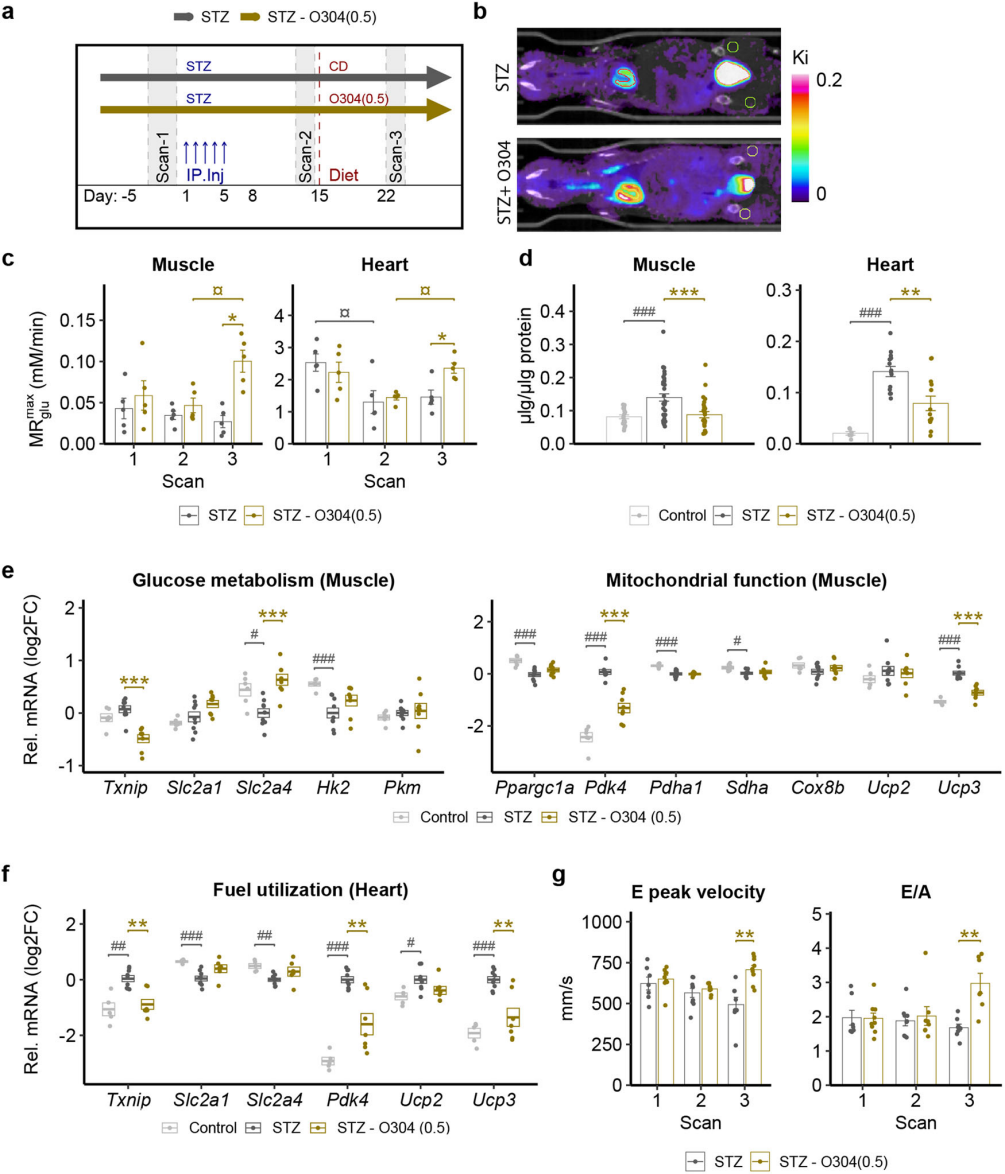
O304 stimulates skeletal muscle glucose uptake and improves cardiac diastolic function in STZ mice. **a** Timeline in days for STZ-treatment and FDG-PET scanning. **b** Representative PET/CT images of FDG uptake in an untreated mouse (upper panel), and an O304-treated mouse (lower panel). On the color scale, warmer colors (yellows or reds) represent higher rates of glucose utilization and cooler colors (blue) lower rates. **c** Estimated muscle and heart maximal glucose uptake (${{MR}}_{glu}^{\max }$) at baseline (Scan 1), 9–10 days after the last STZ injection (Scan 2), and after 1 week of treatment (Scan 3) with 0.5 mg/g O304 (*n* = 5) or no treatment (*n* = 5). **d** Glycogen content in muscle and heart of control (*n* = 6–18) and STZ mice untreated (*n* = 14–36) or treated (*n* = 13–27) with O304 from day 15. **e** Relative mRNA levels of *Txnip*, *Slc2a1, Slc2a4, Hk2, Pkm, Ppargc1a, Pdk4, Pdha1, Shda*, *Cox8b, Ucp2,* and *Ucp3* in muscle of control mice (*n* = 5–7), untreated (*n* = 5–9) and O304-treated (*n* = 6–8) STZ mice. **f** Relative mRNA levels of *Txnip*, *Slc2a1, Slc2a4, Pdk4, Ucp2* and *Ucp3* in heart of control mice (*n* = 5), untreated (*n* = 9) and O304-treated (*n* = 6–7) STZ mice. **g** Transmitral doppler in STZ-treated mice at baseline (Scan 1), 15 days after STZ start (Scan 2,) and after 1 week treatment (Scan 3) with 0.5 mg/g O304 (*n* = 9) or no treatment (*n* = 9). Data are presented as mean ± SEM. Statistical significance between untreated and O304-treated STZ mice was determined by Student’s *t* test (**c**: Muscle, **g**) or by Wilcox-test (**c**: Heart, **d**–**f**) (*P < 0.05, **P < 0.01, ***P < 0.001), and between control mice and STZ mice by Wilcoxon test (**d**–**f**) (^#^*P* < 0.05, ^##^*P* < 0.01, ^###^*P* < 0.001), and between Scans in (**c**, **g**) by one-way repeated ANOVA followed by paired Student’s *t* test (^¤^*P* < 0.05).

O304 has been shown to stimulate insulin independent glucose uptake in vitro in skeletal muscle myotubes^10^ and the potent reduction of glucose levels in O304-treated STZ mice, in the face of ensuing insulin deficiency provide evidence that O304 promotes insulin independent glucose uptake also in vivo. Sequential dynamic [^18^F]-Fluorodeoxyglucose PET analyses of glucose uptake at baseline (scan 1), 9–10 days after the final STZ injection (scan 2), and after 1 week of O304 treatment (scan 3) (Fig. 2a, b and Supplementary Fig. 2), showed that the maximal metabolic rate of glucose, (${{MR}}_{glu}^{\max }$), in skeletal muscle was greatly enhanced in STZ mice following 1 week of O304 treatment (scan 3), compared both with that before treatment (scan 2) and with that of untreated STZ mice at day 22 (scan 3) (Fig. 2c). Moreover, (${{MR}}_{glu}^{\max }$) was reduced in hearts of diabetic STZ mice at day 14–15, i.e., at scan 2, but normalized to baseline levels after 1 week of O304 treatment, i.e., at scan 3, (Fig. 2c). To address the metabolic fate of glucose taken up by skeletal muscle and heart of untreated and O304-treated STZ mice we next analysed skeletal muscle and cardiac glycogen content. Muscle and cardiac glycogen content was reduced in STZ mice treated with O304 compared with untreated STZ mice but increased in muscle and heart of untreated STZ mice compared to that of non-diabetic control mice (Fig. 2d). Thus, under hyperglycemic conditions O304 treatment stimulates glucose utilization rather than glycogen storage.

### O304 promotes gene expression profiles favouring glucose oxidation in muscle and heart of STZ-treated mice

Skeletal muscle *TXNIP* expression levels are negatively correlated with glucose uptake^27,28^ and concurrent with the enhanced skeletal muscle glucose uptake in O304-treated STZ mice, *Txnip* mRNA and protein levels were reduced in skeletal muscle in skeletal muscle of O304-treated compared with untreated STZ mice (Fig. 2e and Supplementary Fig. 3). Moreover, skeletal muscle expression of *Slc2a4*, encoding Glut4, was reduced in STZ mice compared with controls but normalized in O304-treated STZ mice and that of *Slc2a1*, encoding Glut1, tended to be increased (*P* = 0.059) in skeletal muscle of O304-treated STZ mice compared with untreated STZ mice (Fig. 2e). Similarly, the increased expressions of *Pyruvate Dehydrogenase Kinase 4, Pdk4*, a negative regulator of pyruvate dehydrogenase (PDH) and thus oxidative glucose metabolism^33,34^, and *Uncoupling protein 3 (Ucp3)*, which favour lipids as fuel substrate^35^, in STZ mice were reduced in O304-treated mice (Fig. 2e), suggesting that O304 counteracts metabolic inflexibility in skeletal muscle of STZ diabetic mice.

*Txnip* expression was increased also in hearts of STZ mice but comparatively reduced in O304-treated STZ mice (Fig. 2f). Moreover, cardiac expressions of *Slc2a1* and *Slc2a4* were decreased in untreated STZ mice but tended (*P* = 0.088 for both) to be increased in O304-treated mice (Fig. 2f). The expressions of *Pdk4, Ucp2*, and *Ucp3* were increased in heart of untreated STZ mice but attenuated (*P* = 0.055 for *Ucp2*) in O304-treated STZ mice (Fig. 2f). Thus, O304 promoted gene expression changes favouring glucose uptake, oxidative glucose metabolism, and ATP generation in skeletal muscle and heart of STZ mice (Fig. 2e, f). Hyperglycemia alone, i.e., in an insulin deficient and non-obese context, has been shown to rapidly increase cardiac expression of Pdk4 and Ucp3, and to provoke metabolic inflexibility and cardiac dysfunction in mice^36^. Echocardiography analyses revealed that untreated STZ mice showed gradually decreased E peak velocity and increased isovolumetric relaxation time (IVRT), indicating impaired diastolic function, whereas 1 week O304 treatment increased peak E velocity, and thus E/A ratio, and reduced IVRT (Fig. 2g and Supplementary Table 1). The improved filling of the left ventricle in O304-treated mice also resulted in increased stroke volume and cardiac output (Supplementary Table 1). Altogether these findings show that in diabetic STZ mice, O304 ameliorated hyperglycemia by stimulating insulin independent glucose uptake and utilization, mitigated glycogen accumulation, and reverted diabetic cardiomyopathy.

### O304 ameliorates hyperglycemia and promotes gene expression profiles favouring glucose oxidation in muscle of db/db mice

**Fig. 3:**
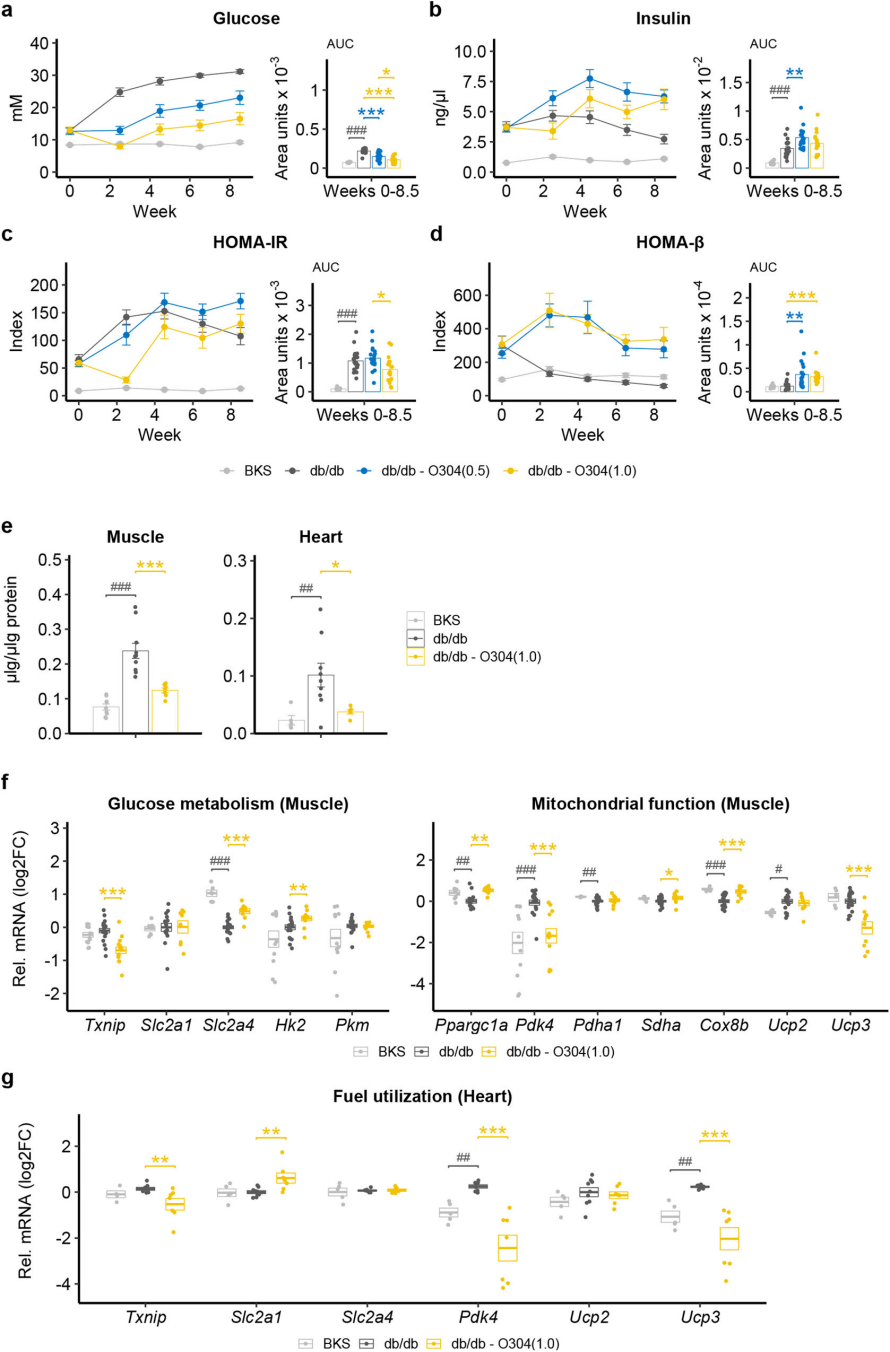
O304 dose-dependently averts hyperglycemia in db/db mice. **a**, **b** Fasted glucose (**a**) and insulin (**b**) levels with area under the curve (AUC) in BKS and db/db mice untreated or treated with 0.5 and 1.0 mg/g O304, respectively (*n* = 15–20/group). **c**, **d** HOMA-IR (**c**) and HOMA-β (**d**) (calculated from (**a**, **b**)) with area under the curve (AUC) in BKS and db/db mice untreated or treated with 0.5 and 1.0 mg/g O304. **e** Glycogen content in muscle and heart of BKS (*n* = 5–9) and db/db mice untreated (*n* = 9–10) or treated with O304 (*n* = 6–7). **f** Relative mRNA levels of *Txnip*, *Slc2a1, Slc2a4, Hk2, Pkm, Ppargc1a, Pdk4, Pdha1, Sdha, Cox8b, Ucp2, and Ucp3* in muscle of BKS (*n* = 6–13) and db/db mice untreated (*n* = 15–22) or treated with 1.0 g/kg O304 (*n* = 8–15). **g** Relative mRNA levels of *Txnip*, *Slc2a1, Slc2a4, Pdk4, Ucp2, and Ucp3* in heart of BKS (*n* = 4–5), untreated (*n* = 8–9) and O304-treated (*n* = 6–7) db/db mice. Data are presented as mean ± SEM. Statistical significance between untreated and O304-treated db/db mice was determined by Welch’s ANOVA followed by Games–Howell post hoc test (**a**–**d**), Student’s *t* test (**e**) or Wilcoxon test (**f**–**g**) (*P < 0.05, **P < 0.01, ***P < 0.001), and between BKS and db/db mice was determined by Wilcoxon test (^#^*P* < 0.05, ^##^*P* < 0.01, ^###^*P* < 0.001) (**a**–**d**, **f**, **g**) or by Student’s *t* test (**e**).

To explore the potential of O304 to ameliorate hyperglycemia in the context of severe insulin resistance, 6 w old male leptin receptor-deficient db/db mice were treated with O304 formulated in the diet at a concentration of 0.5 or 1.0 mg/g O304, denoted O304(0.5) and O304(1.0), respectively, for 9 w. BKS mice were used as controls. At 6 w of age, compensatory hyperinsulinemia was evident in db/db mice and consequently 6 h fasted blood glucose levels were only slightly elevated in db/db mice compared with BKS mice (Fig. 3a, b). As expected, untreated db/db mice failed to compensate for the ensuing insulin resistance and blood glucose levels rapidly increased (Fig. 3a). The increase in blood glucose levels in untreated db/db mice was paralleled by a decline in insulin levels (Fig. 3b), corroborating the β-cell failure phenotype of db/db mice^19,37^. The increase in blood glucose levels were dose-dependently attenuated in O304-treated db/db mice and insulin levels were increased compared both with starting values and with untreated db/db mice (Fig. 3a, b). These findings provide evidence that O304 preserved a compensatory β-cell insulin secretory response in db/db mice. Homeostasis model assessment of insulin resistance (HOMA-IR) and β-cell function (HOMA-β) calculations showed that the decline in β-cell function was attenuated in O304-treated db/db mice despite prevailing insulin resistance (Fig. 3c, d). These data provide evidence that O304 attenuated hyperglycemia in db/db mice largely by preventing β-cell failure and loss of compensatory insulin secretion.

O304 mediated attenuation of hyperglycemia was paralleled by reduced glycogen accumulation in skeletal muscle and heart (Fig. 3e). These findings suggest that O304(1.0) stimulated glucose metabolism in skeletal muscle and heart also in diabetic db/db mice. The decreased expression of *Txnip*, *Pdk4* and *Ucp3* together with the increased expression of *Slc2a4*, *Peroxisome proliferator-activated receptor-gamma coactivator (PGC)−1alpha* (*Ppargc1a*), a positive regulator of mitochondrial biogenesis and respiration, and *Cox8b*, a driver of oxidative phosphorylation, in skeletal muscle of O304-treated compared with untreated db/db mice supports this notion (Fig. 3f). The increased cardiac expression of *Slc2a1* and the attenuated expressions of *Txnip, Pdk4*, and *Ucp3* in treated compared with untreated db/db mice (Fig. 3g) is consistent with that observed in O304-treated STZ mice. Notably, O304 did not increase serum lactate levels in O304-treated STZ and db/db mice (Supplementary Fig. 4). Taken together, these findings provide evidence that, in both STZ and db/db diabetic mice, O304 averts gene signatures associated with metabolic inflexibility, i.e., imbalance between carbohydrate and fat metabolism, which in turn is associated with diabetes and diabetic cardiomyopathy^33,34^.

### O304 stimulates mitochondrial uncoupling in myotubes

**Fig. 4:**
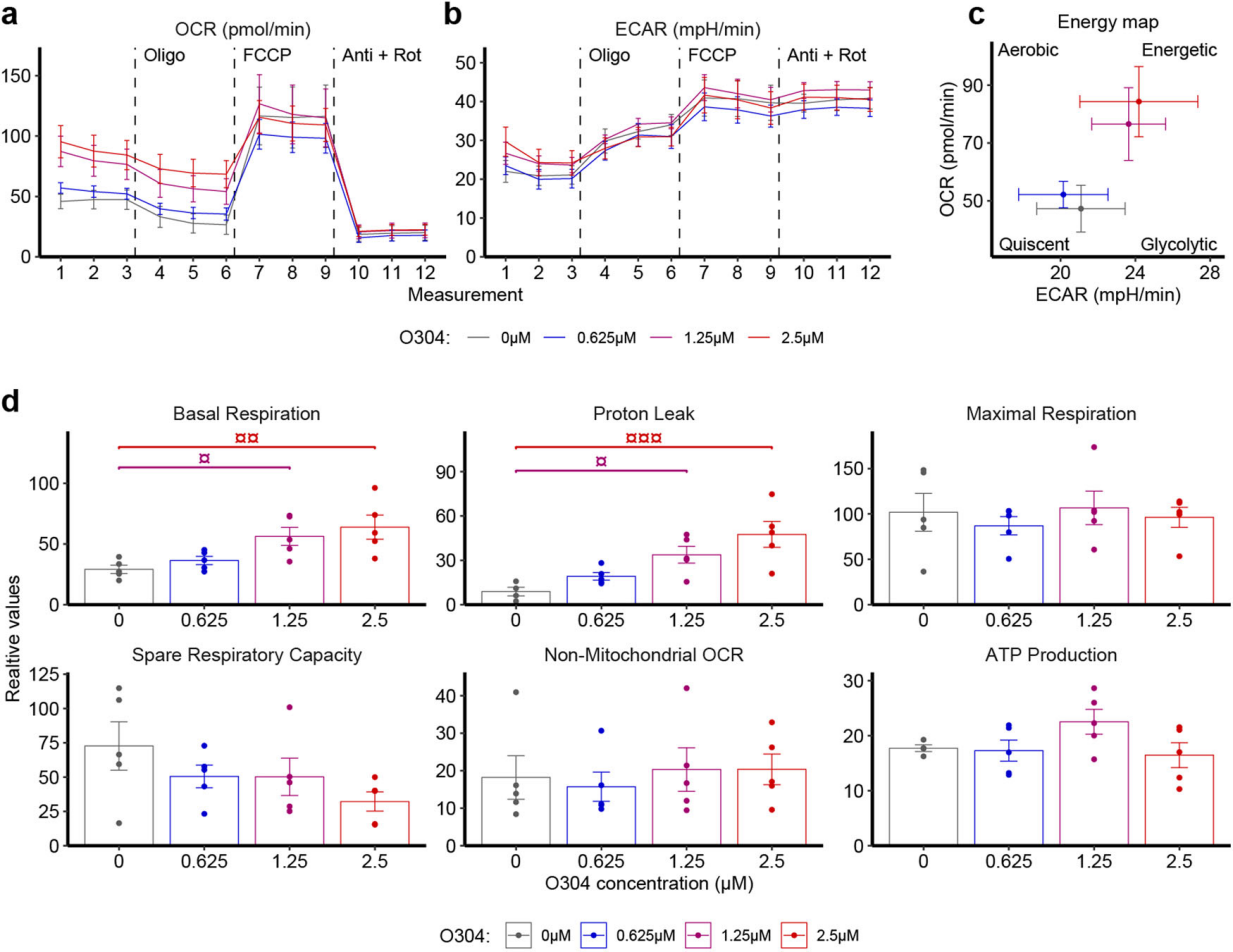
O304 induces mitochondrial uncoupling. **a**, **b** Respirometry plots of intact differentiated C2C12 myotubes +/− 4 h treatment with 0.625, 1.25 and 2.5 μM O304 sequentially injected with oligomycin, FCCP, and a cocktail of rotenone and antimycin showing oxygen consumption rate (OCR) (**a**) and extracellular acidification rate (ECAR) (**b**). **c** OCR vs ECAR plot from last baseline measurement (measurement 3 in (**a**, **b**)). **d** Mitochondrial function parameters calculated from OCR data in (**a**). Data are presented as mean ± SEM. *n* = 5/condition. Statistical significance in d between untreated and O304-treated cells was determined by one-way ANOVA followed by Dunnett’s post hoc test (^¤^*P* < 0.05, ^¤¤^*P* < 0.01, ^¤¤¤^*P* < 0.001).

The increase in glucose utilization and reduced glycogen content observed in skeletal muscle and heart of O304-treated STZ and db/db mice mimics the effects of exercise/contraction^15^, indicating that O304 increases energy expenditure by generating a metabolic demand either via futile cycling and/or mitochondrial uncoupling. To elucidate a potential uncoupling potential for O304, we performed studies of mitochondrial and glycolytic function in intact, differentiated C2C12 myotubes following 4 hours (h) treatment with increasing concentrations of O304. Four hours treatment with O304 dose-dependently increased oxygen consumption rate (OCR), a measure of oxidative phosphorylation in C2C12 myotubes (Fig. 4a). The ratio of basal OCR to basal ECAR (extracellular acidification rate) (Fig. 4a–c), provide evidence that O304 increased the cellular preference for oxidative metabolism. O304 reduction in OCR in response to oligomycin, which blocks the ATP synthase, was similar to that of untreated cells (Fig. 4a, d, which is  consistent both with unaffected ATP production (Fig. 4d) and our previously published findings that O304 did not significantly reduce ATP levels in cells^10^. However, the elevated OCR levels in O304 treated cells in presence of oligomycin provide evidence of proton leak (Fig. 4a, d). Taken together these findings show that O304 increases cellular respiration by functioning as mitochondrial uncoupler, providing evidence that O304, like exercise but unlike ADaM site activators^11,12,15^, induces a metabolic demand that enhances energy expenditure and glucose metabolism.

### O304 preserve β-cell function and mass in db/db mice

**Fig. 5:**
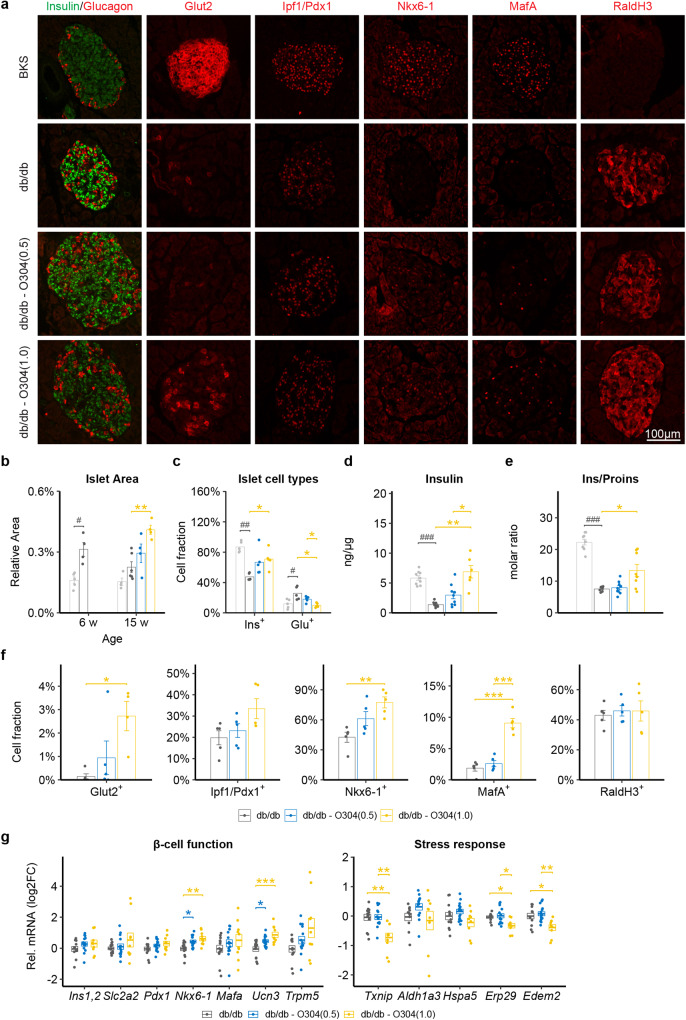
O304 preserves β-cell mass and expression of β-cell markers in db/db mice. **a** Representative immunostaining of pancreases from 15 w old BKS and db/db mice untreated or treated with 0.5 and 1.0 mg/g O304, respectively, for Insulin (green) and Glucagon, Glut2, Ipf1/Pdx1, Nkx6-1, Mafa, and Raldh3 (all red) (*n* = 5/group and antibody). **b** Quantification of islet cell area in 6 w old BKS and db/db mice and in 15 w old BKS and db/db mice untreated or treated with 0.5 and 1.0 mg/g O304, respectively, (*n* = 4–6/group). **c** Insulin (Ins^+^) and glucagon (Glu^+^) positive cell fraction in 15 w old BKS and db/db mice untreated or treated with 0.5 and 1.0 mg/g O304 (*n* = 5 for all groups). **d**, **e** Total pancreatic insulin content (**d**) and pancreatic insulin/proinsulin ratio (**e**) in 15 w old BKS (*n* = 9) and db/db mice untreated (*n* = 7–10) or treated with 0.5 (*n* = 9) and 1.0 mg/g O304 (*n* = 6–8). **f** Quantification of Glut2, Ipf1/Pdx1, Nkx6-1, MafA, and Raldh3 expression in islets of 15 w old db/db mice untreated (*n* = 5) or treated with 0.5 (*n* = 5) and 1.0 (*n* = 4–5) mg/g O304. **g** Relative mRNA levels of *Ins1/2*, *Slc2a2, Pdx1, Nkx6-1*, *Mafa*, *Ucn3, Trpm5, Txnip, Aldh1a3, Hspa5, Erp29*, and *Edem2* in islets of 15 w old db/db mice untreated (*n* = 11–13) or treated with 0.5 (*n* = 14–15) and 1.0 (*n* = 9–10) mg/g O304. Data are presented as mean ± SEM. Statistical significance between untreated and O304-treated db/db mice was determined by Welch’s ANOVA followed by Games–Howell post hoc test (**b**–**f**) or Kruskal–Wallis test followed by Dunn’s post hoc test (**g**) (*P < 0.05, **P < 0.01, ***P < 0.001), and between BKS and db/db mice was determined by Wilcoxon test (**b**–**e**) (^#^*P* < 0.05, ^##^*P* < 0.01, ^###^*P* < 0.001).

Consistent with the described initial compensatory increase in β-cell mass in db/db mice^19,37^, islet cell area was increased at 6 w of age in db/db compared with BKS mice (Fig. 5a, b). By 15w of age islet cell area was relatively reduced (*P* = 0.09) compared with that at 6 w of age in untreated db/db mice while islet cell area was dose-dependently preserved in the O304-treated mice (Fig. 5a, b). The percentage of Ins^+^ cells was also increased in O304-treated compared with untreated db/db mice whereas cells positive for other pancreatic endocrine hormones tended to be reduced in O304-treated db/db mice (Fig. 5c and Supplementary Fig. 5a). Thus, O304 preserved β-cell mass in db/db mice. Consistently, pancreatic insulin and proinsulin content was increased in O304-treated db/db mice compared to untreated db/db mice (Fig. 5d and Supplementary Fig. 5b). Moreover, pancreatic insulin:proinsulin ratio, a measure of the efficiency of proinsulin processing to insulin that is perturbed in β-cells of type 2 diabetics, was increased in db/db mice treated with the highest, 1 mg/g, dose of O304 compared with untreated db/db mice (Fig. 5e). Together these data provide evidence that O304 both averts β-cell loss and preserves β-cell function in db/db mice.

Protein expression of the glucose transporter Glut2, encoded by *Slc2a2*, was partially restored in islets of O304-treated db/db mice (Fig. 5a, f). The expressions of the transcription factors Nkx6.1 and MafA, markers of a mature β-cell identity, were increased and Ipf1/Pdx1 tended to be increased in islets of O304-treated db/db mice (Fig. 5a, f). mRNA expression of Raldh3, encoded by *Aldehyde dehydrogenase 1a3* (*Aldh1a3)*, which is upregulated in diabetic islets^38^, was however not altered (Fig. 5a, f). mRNA expression analyses of isolated islets showed that the expression of *Urocortin 3 (Ucn3)*, a marker of mature β-cells and regulator of insulin secretion^39^, was increased in islets from O304-treated db/db mice (Fig. 5g). The expressions of the transcription factors *Ipf1/Pdx1* and *Mafa* were largely unaltered whereas that of *Nkx6.1* was increased in islets of O304-treated db/db mice as compared with untreated db/db mice (Fig. 5g). O304 treatment did not change the expression of *Aldh1a3* (Fig. 5g). The expression of *Txnip*, which negatively affects insulin transcription and provokes oxidative stress in β-cells^31^, was reduced in islets of db/db mice treated with the highest, 1 mg/g, dose of O304 compared with untreated db/db mice. Similarly, the expressions of the ER-stress associated genes *Endoplasmic Reticulum Protein 29 (Erp29)* and *ER degradation-enhancing alpha-mannosidase-like 2 (Edem2)* were reduced in islets of db/db mice treated with the highest, 1 mg/g, dose of O304, whereas that of *Heat Shock Protein Family A Member 5 (Hspa5)*, encoding Bip, was unaltered (Fig. 5g). These data suggest that reduced expression of *Txnip* and ER stress genes, together with increased expression of transcription factors and genes regulating β-cell identity and insulin secretion, contribute to the O304 mediated maintenance of compensatory β-cell function, GSIS, and β-cell mass in insulin resistant db/db mice. The maintained compensatory insulin secretion in O304-treated db/db mice likely explain the unchanged expression of *Aldh1a3*, suggesting that increased *Aldh1a3* expression is a marker for metabolically challenged β-cells.

### O304 alleviates islet gene expression changes provoked by acute hyperglycemia

**Fig. 6:**
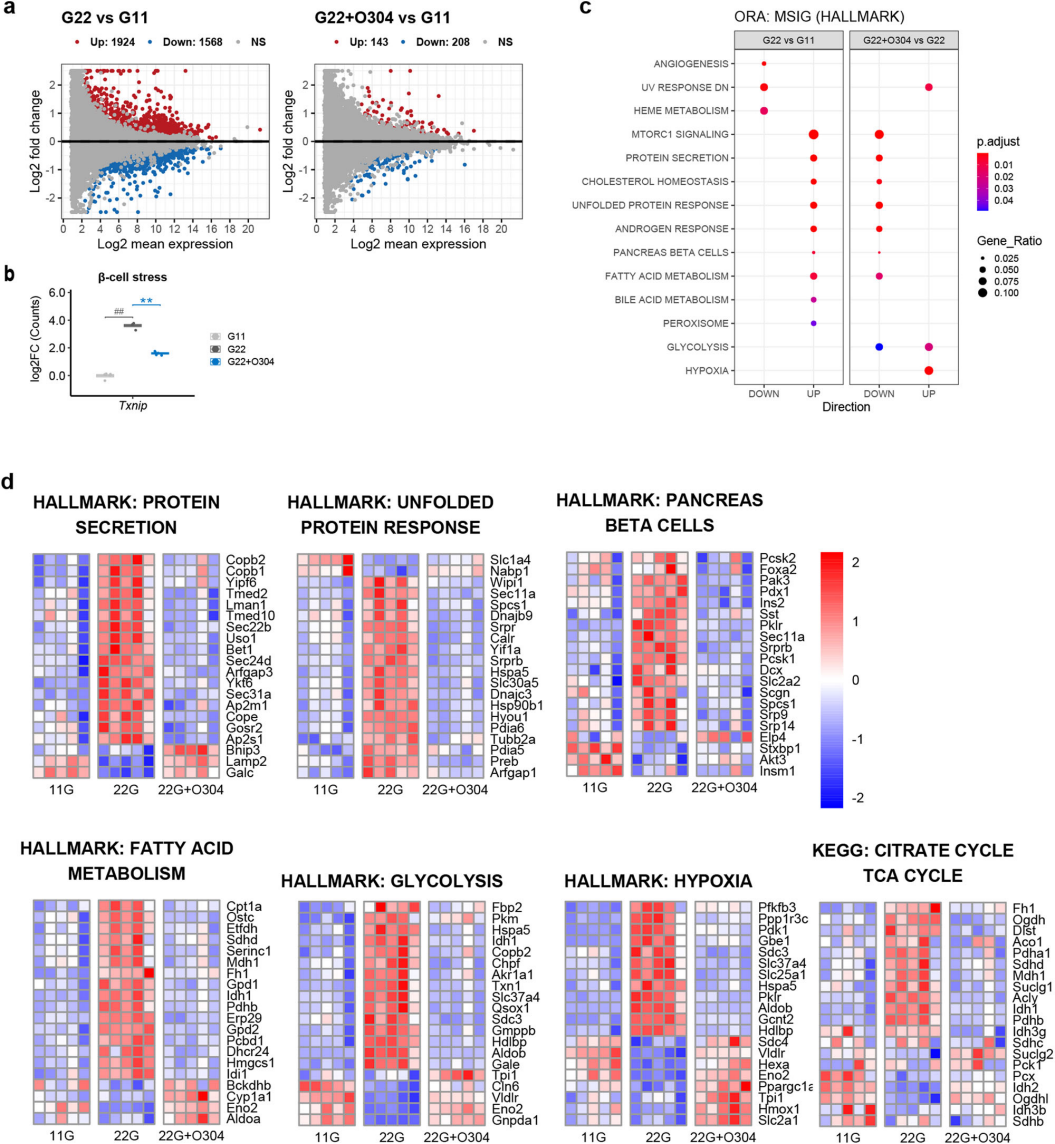
O304 treatment mitigates hyperglycemia induced islet gene expression changes in ex vivo cultured islets. **a** MA plots showing differentially expressed genes between mouse islets cultured at 22 mM (G22) vs 11 mM (G11) glucose and 22 mM glucose + 5 μM O304 (G22 + O304) vs 11 mM glucose. **b** Log2 fold-change of normalized read counts for Txnip in mouse islets cultured at 11 mM glucose, 22 mM glucose, and 22 mM glucose + 5 μM O304 (n = 5 for all groups). **c** Overrepresentation analysis (ORA) of Molecular signature Hallmark gene sets (MSIG) in mouse islets cultured at 22 mM vs 11 mM glucose and 22 mM glucose + 5 μM O304 vs 22 mM glucose. Data are presented as mean ± SEM. Statistical significance was determined by Wilcoxon test for 22 mM vs 11 mM glucose (^##^*P* < 0.01) and for 22 mM glucose + 5 μM O304 vs 22 mM glucose (*P < 0.05, **P < 0.01). **d** Heatmaps of normalized read counts of DEGs from enriched categories from (**b**) and from KEGG: TCA_CYCLE. The number of genes is limited to the 20 most significant DEGs. In this comparison, deepening colors represent progressively lower (blue) or higher (red) mRNA levels for any given gene.

To investigate the ability of O304 to directly modulate the response of pancreatic islets to glucotoxic conditions we next performed RNAseq analysis of isolated mouse and human islets cultured ex vivo at low (11 mM) and high (22 mM) glucose +/− 5 μM O304. Mouse islets responded to high glucose by upregulating 1924 and downregulating 1568 differentially expressed genes (DEGs) (Fig. 6a and Supplementary Fig. 6a–c). At high glucose O304 reduced the number of DEGs to 143 upregulated and 208 downregulated (Fig. 6a and Supplementary Fig. 6a–c). As expected, exposure of mouse islets to high glucose resulted in a significant increase in *Txnip* expression whereas O304 attenuated the increase in *Txnip* expression at high glucose (Fig. 6b). Overrepresentation analysis (ORA) of Molecular signature Hallmark, KEGG and Wiki-pathways gene sets^40^ revealed that the gene sets associated with enhanced beta-cell activity such as “Protein Secretion”, “Unfolded protein response” and “Pancreas beta-cells”, were overrepresented among DEGs upregulated by high glucose but averted by O304 at high glucose (Fig. 6c). The metabolic pathways “Fatty acid metabolism”, “Glycolysis” and “Hypoxia”, were also overrepresented together with “KEGG: TCA cycle” (*P* = 0.073), among DEGs regulated by high glucose and counter regulated by O304. Hierarchal clustering of the top 20 most significant DEGs of these pathways, regardless of direction, showed opposing regulation by high glucose and addition of O304 (Fig. 6d). These results demonstrate the ability of O304 to directly preserve key metabolic gene expression patterns in β-cells under glucotoxic conditions. Gene expression differences in human islets were dominated by donor effects, and even after batch correction, few DEGs were detected (Supplementary Fig. 6d–f). Nonetheless, gene set enrichment analysis (GSEA) show that high glucose induced genes within the “Pancreatic beta-cells” gene set, which was counter regulated by O304, and that alike that observed for mouse islets, O304 induced upregulation of genes within “Hypoxia” and “Glycolysis” gene sets (Supplementary Fig. 6g). These results suggest that O304 ameliorates metabolic adaptations of β-cells to hyperglycemia and may thereby contribute to β-cell rest and preservation of β-cell function under overt diabetic conditions.

### O304 prevents the negative effects of hyperglycemia on GSIS in INS-1E cells

**Fig. 7:**
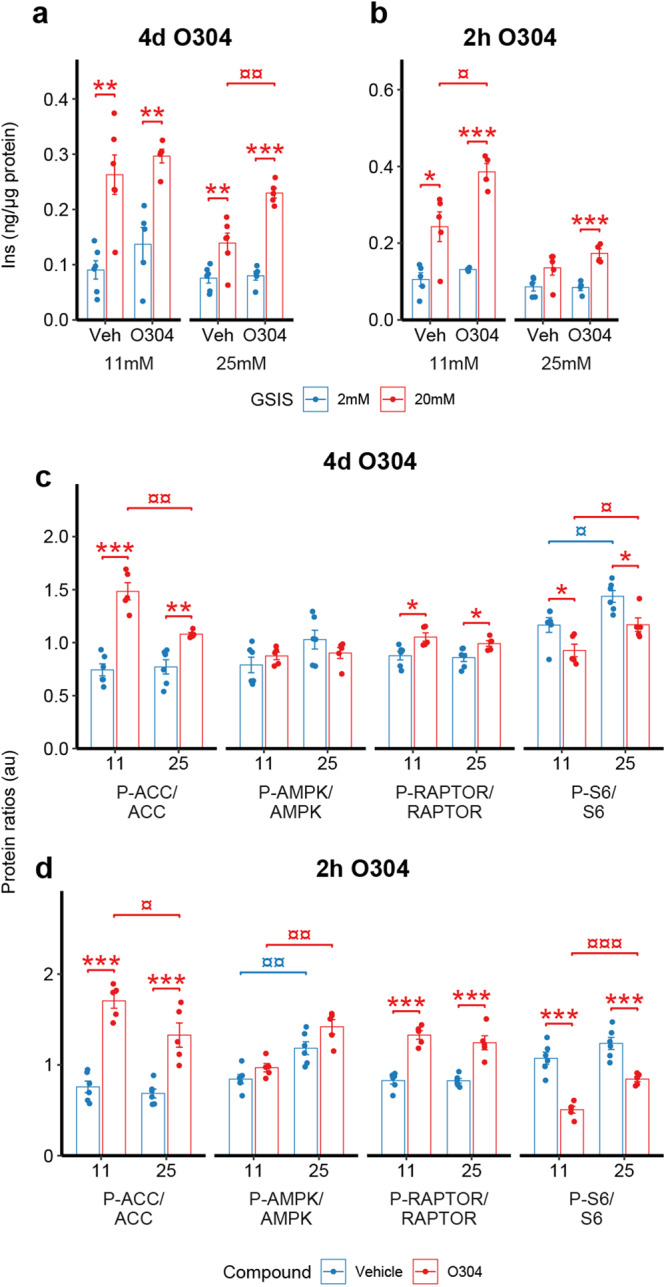
O304 mitigates the effect of chronic hyperglycemia on GSIS, mTORC1, and AMPK signaling in INS-1E cells. **a**, **b** GSIS of INS-1E cells cultured under 11 mM or 25 mM glucose for 4 d, untreated and treated with 5 μM O304 for 4 days (**a**) (*n* = 5–6/group) or 2 h (**b**) (*n* = 4–5/group). **c**, **d** Protein expression ratios of Phosphorylated (P-) and total ACC, AMPK, RAPTOR, and S6 in INS-1E cultured under 11 mM or 25 mM glucose for 4 d, untreated (*n* = 6) and treated (*n* = 5) with 5 μM O304 for 4 days (**c**) or 2 h (**d**). Data are presented as mean ± SEM. Statistical significance was determined by two-way ANOVA followed Tukey’s post hoc test between untreated and O304-treated cells (*P < 0.05, **P < 0.01, ***P < 0.001) and 11 mM and 25 mM glucose cultured cells (^¤^*P* < 0.05, ^¤¤^*P* < 0.01).

A shift from mTORC1 to AMPK signalling is critical for the functional maturation of β-cells and a reverse switch from AMPK to mTORC1 signalling has been demonstrated in T2D β-cells^41^. Moreover, recent findings showed that chronic hyperglycemia activates mTORC1 signalling in β-cells in vitro, resulting in reduced glucose metabolism and impaired GSIS^42^. Our RNAseq analysis identified mTORC1 signalling to be upregulated in islets exposed to high (22 mM) glucose and that O304 prevented the increase in mTORC1 signalling at high glucose (Fig. 6c). To explore the functional consequences of O304 on GSIS under conditions of chronic hyperglycemia we cultured INS-1E cells at 25 mM glucose for 4 days (d). In agreement with the findings of ref. ^42^, 4d culture at 25 mM glucose impaired GSIS, concomitant exposure to O304 during the entire 4 d culture period averted however these negative effects (Fig. 7a, b). Short term (2 h) exposure of INS-1E cells to O304 at the end of the 4d culture period also partly reversed the negative effects of chronic hyperglycemia on GSIS (Fig. 7b). Thus, O304 both prevented and reverted the negative effects of chronic hyperglycemia on GSIS from INS1-E cells. Analyses of downstream targets of mTORC1, i.e., ribosomal protein 6 (pS6), and AMPK, i.e., Acetyl-CoA carboxylase (ACC) and Raptor, showed that both long- and short-term exposure of INS1-E cells to O304 reduced pS6-Ser240/244 levels but increased pACC-Ser79 and pRaptor-Ser792 levels (Fig. 7c, d and Supplementary Fig. 7). Together, these findings provide evidence that O304 preserves β-cell function under hyperglycemic conditions by antagonizing mTORC1 signalling and preserving AMPK signalling.

## Discussion

Current pharmacological treatment of T2D target either glucose output from the liver, glucose reuptake in kidney, or stimulates insulin secretion from already stressed β-cells^43^. No diabetes drug in clinical use increases glucose uptake/utilization in skeletal muscle or mitigates β-cell failure^44^. Here, we assessed the effect of the clinical stage, AMPK activator O304 with respect to both skeletal muscle glucose uptake and β-cell function in diabetic mice. We provide evidence that O304 ameliorates diabetes by stimulating glucose effectiveness, an underappreciated and largely ignored contributor to glucose disposal^5,45^. Moreover, alike exercise, O304 increased glucose utilization and prevented glycogen accumulation, likely by acting as a mitochondrial uncoupler that creates a metabolic demand. Additionally, O304 preserved β-cell function in an insulin resistant, hyperglycemic context in vivo and prevented, and reverted, the negative effects of chronic hyperglycemia on β-cell function/GSIS in vitro, at least in part by antagonizing mTORC1 signalling.

In STZ mice O304 reduced hyperglycemia by stimulating insulin independent glucose uptake and utilization in skeletal muscle, i.e., stimulated glucose effectiveness. Insulin-independent glucose uptake is mediated both through Glut1^46^ and, under conditions of exercise/contraction, Glut4^47,48^. TXNIP levels have been shown to be inversely correlated with both GLUT1 and GLUT4 levels^27–29^ and in addition *Slc2a4* expression has been shown to be reduced by hyperglycemia^49^. O304 reduced *Txnip* expression and stimulated primarily *Slc2a4* and to lesser extent *Slc2a1* expression in skeletal muscle under both insulin deficient and insulin resistant conditions, suggesting that the increased expression of these glucose transporters together contributes to the insulin independent uptake of glucose in skeletal muscle of STZ mice. Similarly, reduced expression of *Txnip* together with the tendency of increased *Slc2a1 and Slc2a4* expression in hearts of O304-treated STZ mice correlates with enhanced cardiac glucose uptake in these mice.

Under conditions of severe insulin resistance or insulin deficiency, glucose uptake is still, via the mass action effect of glucose, enhanced in response to elevated glucose levels^4,5^ and the intracellular pool of glucose has been reported to be increased in hearts of patients with T1D suggesting that glycolysis, rather than glucose uptake, is impaired in these patients^50^. Moreover, although glucose effectiveness has been reported to be reduced in T1D and T2D subjects, referred to as glucose resistance^9^, it still contributes to glucose disposal also in these subjects, and glucose effectiveness has been suggested to compensate for defects in insulin mediated glucose disposal and glycogen synthase activation in skeletal muscle of T2D individuals^51,52^. Consequently, a mass action driven uptake of glucose in a context of reduced glucose metabolism, as suggested by the muscle and cardiac gene expression profiles in untreated STZ and db/db diabetic mice, may together contribute to the increase in glycogen content observed in muscle and heart of these mice compared with non-diabetic mice. Notably, increased heart glycogen content and a tendency to increased skeletal muscle glycogen levels in diabetic rodents was observed also by Mellor and co-workers^53^.

AMPK has been shown to regulate insulin independent skeletal muscle glucose uptake following exercise^54^, implying enhanced glucose effectiveness post-exercise. However, stimulation of glucose uptake by ADaM site pan-AMPK activators increases glucose uptake but not subsequent utilization, resulting in glycogen accumulation in skeletal muscle and heart^11,12,15^, hindering the clinical use of these such compounds. O304 enhances glucose uptake and utilization without increasing muscle or heart glycogen content. Exercise/contraction stimulates skeletal muscle glucose uptake and, by generating a metabolic demand/increase in ADP, promotes increased TCA flux and glucose oxidation to replenish ATP, thereby preventing glycogen accumulation^15^. O304 acts as a mitochondrial uncoupler in myotubes and as mitochondrial uncoupling generates a metabolic demand/ADP the function as a mitochondrial uncoupler provides a mechanistic explanation by which O304 stimulates glucose utilization instead of glycogen accumulation. An uncoupling effect of O304 is also consistent with our previous data showing that O304 increased oxygen consumption and energy expenditure in vivo in DIO mice and potently both prevented and reduced obesity. Thus, the metabolic fate of glucose following stimulation of skeletal muscle uptake differ between exercise/O304 and ADaM site AMPK activators. Notably, AMPK activation is an integral part of mitochondrial uncoupling. Mitochondrial uncoupling generates ADP, which will transiently increase pAMPK, and the availability of ADP is the most important factor in determining the rate of oxidative phosphorylation and increased glucose and fatty acid oxidation will replenish ATP levels to reduce pAMPK levels. Moreover, AMPK is required for both basal OCR and for uncoupling induced OCR^55,56^. O304 directly suppresses the dephosphorylation of pAMPK^10^ and as a mitochondrial uncoupler O304 would additionally indirectly increase pAMPK. These dual functions of O304 may be highly favorable in promoting both glucose uptake and glucose utilization in T2D patients.

Increased expression of *Pdk4* in skeletal muscle and heart leads to inhibition of PDH, i.e., the rate limiting enzyme of glucose oxidation, which in turn leads to metabolic instability with mitochondrial dysfunction and hyperglycemia as result^33^. *Pdk4* expression has been shown to be increased in skeletal muscle and heart of obese, insulin resistant, and diabetic animal models, and to be associated with insulin resistance and cardiac dysfunction^33,34^. The decreased expression of *Pdk4* and *Ucp3* in skeletal muscle of O304-treated, compared with untreated, STZ and db/db mice provide evidence that O304 attenuates diabetic induced metabolic inflexibility and stimulates oxidative glucose metabolism in skeletal muscle. Moreover, metabolic inflexibility has been associated with development of diabetic heart failure^34,57^. Consistently, diabetic STZ mice developed hyperglycemia associated diabetic cardiomyopathy that was potently averted by treatment with O304 for 1 week. Combined, the decreased cardiac expressions of *Pdk4* and *Ucp3*, reduced cardiac glycogen content, and reversion of diabetic cardiomyopathy in O304-treated STZ mice provide evidence that O304 restores cardiac metabolic flexibility and glucose oxidation, i.e., cardiac efficiency, in these mice.

Our study also provide evidence that O304 treatment preserves the β-cell compensatory response in db/db mice by partly alleviating β-cell stress as judged by the reduced expression of *Txnip* and ER-stress associated genes in β-cells of O304-treated db/db mice. Perturbed proinsulin processing is a hallmark of β-cell dysfunction^58^ and has been suggested to be a consequence of enhanced secretory demand that impairs the granule retention of proinsulin required for processing to insulin^59^. The increased ratio of insulin to proinsulin, i.e., improved proinsulin processing, observed in islets of O304-treated compared with untreated db/db mice likely reflects the reduction of hyperglycemia and amelioration of β-cell stress in O304-treated mice. HOMA-β calculations also show upon a preservation of β-cell function in O304-treated compared with untreated db/db mice in which HOMA-β rapidly declined. O304 mediated preservation of β-cell function was evident despite prevailing insulin resistance, providing evidence that amelioration of β-cell stress by O304 is direct and not secondary to alleviation of insulin resistance.

A direct role for O304 in preserving a functional β-cell identity is coherent with our previous findings that O304 reduced β-cell stress and amyloid formation in islets ex vivo cultured at high glucose levels^10,60^. Moreover, our RNAseq analyses of islets exposed to acute hyperglycemia ex vivo show that O304 mitigates the immediate gene expression adaptions of β-cells to glucotoxic conditions, including increased mTORC1 signalling and *Pdk1* expression. Recent findings by Ashcroft et al., showed that chronic hyperglycemia increased mTORC1 signalling with concomitant reduction of AMPK signalling in β-cells, which leads to increased expression of *Pdk1* that through inhibition of PDH limits the entry of pyruvate into the citric cycle and thus negatively affects oxidative phosphorylation and insulin secretion^42^. O304 counteracted mTORC1 signalling and preserved GSIS under conditions of chronic hyperglycemia in vitro. Taken together, these findings provide evidence that O304 ensures β-cell rest and preserves β-cell function under hyperglycemic conditions by reducing mTORC1 activity, and thus *Pdk1* expression, and maintaining AMPK activity.

Although we previously showed that O304 stimulated insulin independent glucose uptake in vitro in differentiated myotubes in an AMPK dependent manner^10^, a role for AMPK in O304 stimulation of glucose uptake/utilization and preservation of β-cell function in vivo remains to be explored. Notably, mitochondrial uncoupling transiently increases ADP, which is the most important factor in determining the rate of oxidative phosphorylation. ADP will increase pAMPK, and AMPK activity may be required for both basal OCR and for uncoupling induced OCR^55,56^. Thus, a transient increase in pAMPK is an integral part of uncoupling, and the combined effects of O304 as a mitochondrial uncoupler and suppressor of pAMPK dephosphorylation may synergistically act to increase AMPK activity, and importantly also generate a metabolic demand (Supplementary Fig. 8). Moreover, the recent identification of the ADP/ATP carrier (AAC) as a mitochondrial uncoupling protein present in all cells^61^ prompts future studies on the role for AAC in mediating the in vivo metabolic effects of O304. Thus, the relative contribution of O304 as an AMPK activator and/or mitochondrial uncoupler in stimulation of glucose uptake/utilization and preservation of β-cell function in vivo will require future analyses in mice with conditional inactivation of AMPK and AAC in muscle and beta-cells, respectively.

In conclusion, our findings provide evidence that O304 exerts dual anti-hyperglycemic effects manifested as stimulation of glucose effectiveness and glucose utilization in insulin deficient settings, and by preserving β-cell function in an insulin resistant/hyperglycemic context. The dual function of O304 as an AMPK activator and mitochondrial uncoupler (Supplementary Fig. 8), and the unique dual, anti-diabetic properties exerted by O304 holds great potential for treatment of diabetes provoked both by insulin resistance and by insulin deficiency.

## Methods

### Animals and diets

Animal experiments were approved by the Animal Review Board at the Court of Appeal of Northern Norrland in Umeå (approval numbers A4-19 and A12-20) and conducted in accordance with Guidelines for the Care and Use of Laboratory animals. In all, 5–6 weeks old Leptin receptor-deficient male BKS.Cg-Dock7m +/+ Leprdb/J (db/db), #000642, and male C57BLKS/J (BKS), #000662, mice were obtained from The Jackson Laboratory, USA. F1 mice were obtained from breeding male C57BL/6J mice (#000664, The Jackson Laboratory, USA) with female CBA/CaCrl (#609, Charles River, UK). In all, 9–11 weeks old male F1 mice were treated with multiple low dose streptozotocin (50 mg/kg*day for 5 consecutive days; freshly prepared in 0.1 mM sodium citrate, pH 4.5) to induce diabetes. Mice were ad libitum fed D10001 diet (Research diets, Inc, New Brunswick, NJ) or D10001 formulated with O304 at 0.25 mg/g, 0.5 mg/g and 1 mg/g O304 (CAS # 1261289-04-6, kindly provided by Betagenon AB, Umeå, Sweden). Cohorts of BKS, db/db and F1 mice were housed in groups of 4–5 mice/cage. Mice with apparent health problems such as >10% reduction in body weight or fighting were excluded with no differences between groups. In the different cohorts, mice were randomly allocated to the cages, based on weight and fasting blood glucose and allocated cage-wise or, if possible, individually to different treatments in order to minimize influence of starting weight and glucose homeostasis. For in vitro analyses such as western blot, qPCR, histology and immuno-histology work, samples from 5–9 mice/diet were randomly selected. All in vivo analyses were performed between 9 am to 3 pm.

### Glucose and serum related measurements

Blood glucose was measured using a Glucometer (Accu-Chek Aviva, Roche, Sweden) and plasma insulin was analyzed using the ultrasensitive mouse insulin ELISA kit (Chrystal Chem Inc. #90080). The homeostasis model for insulin resistance (HOMA-IR) was calculated via:

1 $${{{\rm{HOMA}}}}{\mbox{-}}{{{\rm{IR}}}} = \frac{{{{\rm{fasting}}}}\, {{{\rm{blood}}}}\, {{{\rm{glucose}}}} \,({{{\rm{mmol/L}}}}) \times \,{{{\rm{fasting}}}}\, {{{\rm{plasma}}}}\, {{{\rm{insulin}}}}\, ({{{\rm{\mu}}}} {{{\rm{U/mL}}}})}{22.5}$$

2 $${{{\rm{HOMA}}}}{\mbox{-}}{{{\rm{\beta}}}} = \frac{20 \times \,{{{\rm{fasting}}}}\, {{{\rm{plasma}}}}\, {{{\rm{insulin}}}}\, ({{{\rm{\mu}}}} {{{\rm{U/mL}}}})}{{{{\rm{fasting}}}}\, {{{\rm{blood}}}}\, {{{\rm{glucose}}}} \,({{{\rm{mmol/L}}}}) -\!3.5}$$

### PET analyses of in vivo glucose uptake

Each mouse was subjected to three dynamic positron-emission tomography (PET)-scans, at baseline, 9–10 days after the last STZ injection, and after 1 week of O304 treatment. A dynamic (frames 8 × 30 s, 8 × 60 s, 6 × 180 s, 2 × 300 s) 40-min PET acquisition commenced with the injection of 50–70 µL of 13.9 ± 2.3 MBq [^18^F]-Fluorodeoxyglucose (FDG) dissolved in saline. The maximal metabolic rate of glucose, (${{MR}}_{glu}^{\max }$), was calculated using Patlak analysis of PET data combined with measured glucose concentrations. Mice were starved for three hours before each scan. Before the scan, mice were sedated with <2% isoflurane (Attane VET, VM Pharma, Stockholm, Sweden) in oxygen (800 mL/min) and cannulated via the tail vein using a 27 G needle and a tailor-made catheter. PET/CT (nanoScan PET/CT, Mediso, Hungary) imaging started with a 50 kV, 0.088 mAs, helical CT acquisition reconstructed to images with 0.375 × 0.375 × 0.377 mm^3^ voxel size. PET images were reconstructed to a voxel size (0.4 × 0.4 × 0.4), with 4 iterations and 4 subsets using the Tera-Tomo 3D iterative reconstruction, with attenuation, scatter, and randoms-correction. Image analysis and Patlak pharmaco-kinetic calculation was performed with imlook4d software (https://github.com/JanAxelsson/imlook4d). During the scans, mice were supervised on a temperature-controlled bed, and blood glucose was measured at 10, 20, and 40 min after injection (Accu-Chek Aviva, Roche, Sweden), using tail vein blood. A region-of-interest (ROI) consisting of both the left and right gluteus medius/rectus femoris muscles was defined in coronal views as 10-pixel-diameter and 10-slice cylinders. The myocardial ROI was defined by thresholding a manually delineated search volume in the last PET frame at 40% of the highest voxel. The vena cava ROI was defined as the voxels above 60% of the maximum voxel in the first frame with uptake. An image-derived input function was approximated using the time-activity curve from the vena cava ROI. This is a slight simplification compared to an arterial input function in the early time frames but due to the manual injection we could not obtain a reliable arterial blood peak in any anatomical region nor in the left ventricle due to spill-in from the early uptake in the heartmuscle^62^.

Patlak analysis^63^ was performed employing above image-derived input function, determining the irreversible uptake rate *K*_*i*_ by linear regression between 16 and 40 min. Patlak *K*_*i*_ values were calculated on ROI level for quantification, and on voxel level for illustrations. The saturated glucose consumption (${{MR}}_{{glu}}^{\max }$), was estimated^64^ as

3 $${{MR}}_{{glu}}^{\max }={K}_{i}\left({K}_{m}+{C}_{{glu}}\right)$$

with the Michaelis–Menten *K*_*m*_ = 130 mg/dL, using the average measured blood glucose level *C*_*glu*_ from the three samples taken over the scan.

### Echocardiography

Transthoracic echocardiography was performed on db/db (*n* = 18) mice and STZ-treated (*n* = 18) mice, using the Vevo 3100 system and the MX550D transducer (Fujifilm VisualSonics, Toronto, ON, Canada). 1.5–2% isoflurane (Attane Vet, VM Pharma Stockholm, Sweden), in 0.8 L min^−1^ O_2_ (g) was used for anaesthesia. In short; mice were sedated and placed on a temperature-controlled table, chest hair was removed using hair removal cream. Respiration and ECG were monitored during the scan, and anaesthesia adjusted to avoid depression of respiration. Total scan time did not exceed 15 min. Stroke volume, cardiac output and wall thicknesses were measured in the parasternal long-axis view using B-mode and M-mode images. Diastolic left ventricle inflow used transmitral doppler in the apical four-chamber view. Off-line analysis used Vevo LAB software 5.6.1 (Fujifilm VisualSonics, Toronto, ON, Canada), and was done in a blinded manner.

### Pancreas isolation, preparation, and immunohistochemistry

Pancreases were isolated for immunohistochemical analysis, acid ethanol extraction, and insulin measurements essentially as described^65^. In brief, total pancreatic insulin was extracted using acid ethanol (75% EtOH, 0.2 M HCl) and measured by ELISA (Mercodia #10-1249-01). For immunohistochemistry pancreatic tissue was fixed in 4% paraformaldehyde in 0.1 M sodium-phosphate buffer pH 7.4 for 1–2 h at 4 °C, washed in TBST (50 mM Tris-HCI pH 7.4, 150 mM NaCl, 0.1% Triton X-100) and transferred to 30% sucrose in PB and incubated for 24 h at 4 °C before freezing, sectioning and immunostaining. Primary and secondary antibodies used for immunohistochemistry are listed in Supplementary Table 2. Tissue slides were blocked with TBST 1 hr at RT before incubation with antibodies diluted in TBST + 10% fetal calf serum. Total pancreatic islet area was determined using a cocktail of insulin, glucagon, and somatostatin antibodies.

### Glycogen determination

Gastrocnemius muscle and left cardiac ventricle glycogen content was determined using a Glycogen Assay Kit (Abcam #ab65620) according to the manufacturer’s recommendations.

### Cell lines

INS-1E (AddexBio #C0018009) and C2C12 (ATCC CRL-1772) were commercially available and therefore not authenticated following purchase. The cells were free of mycoplasma as determined by PCR.

### Mitochondrial respiration assays

C2C12 myoblasts were obtained from ATCC (CRL-1772) and maintained in growth media (DMEM, Gibco #41966-029), 10% fetal bovine serum (FBS, Gibco #10500-064) and 20U/ml Penicillin-Streptomycin (Gibco #15140-122). To obtain myotubes, C2C12 myoblasts were seeded in poly-l-Lysin coated XF96 plates at 7500 cells/cm^2^, cultivated in growth media for 3 days to 80% confluence, thereafter switched to differentiation media (DM; growth media with FBS replaced by 2% horse serum (ThermoFisher #26050070) for 6 days with media changes every 2 days. Respiration assays were performed using a Seahorse XFe96 Extracellular flux analyzer (Agilent) according to the manufacturer’s “Mito-stress” protocol and mitochondrial parameters. Briefly, differentiated myotubes were pre-treated by switching to growth media with 1% horse serum together with O304 (sodium salt formulation) for 4 h. Myotubes were then equilibrated for 1 h in Seahorse assay medium (Seahorse Base Medium (Agilent # 103335-100, 1 mM Na-pyruvate, 10 mM glucose, 2 mM l-glutamine) adjusted to pH 7.4 and supplemented with O304 as during pre-treatment. Measurements of oxygen consumption rate (OCR) and extracellular acidification rate (ECAR) was collected for baseline, followed by sequential addition of 1 μM oligomycin, 2 μM FCCP, and 0.5 μM rotenone + 0.5 μM antimycin A. Mitochondrial function parameters were calculated according to the manufacturer’s recommendations.

### Islet isolation and culture

Mouse islets were isolated essentially as described^66^. Briefly, the pancreas was retrogradely filled with a 0.7 mg/ml Collagenase P solution dissolved in Hanks balanced salt solution (HBSS) without CaCl2 and MgCl_2_ (GIBCO #14180). The pancreas was subsequently removed and incubated in the same solution for 10–15 min at 37 °C. The incubation was stopped by addition of HBSS with CaCl_2_ and MgCl_2_ (GIBCO #14060). After rinsing, the islets were handpicked under a stereomicroscope and cultured for 48 h in media (RPMI 1640 medium [GIBCO #11879-0], 1% fetal bovine serum [GIBCO #10500-064], 10 mM HEPES [Umeå University, Laboratory medicine], 1 mM sodium pyruvate [Gibco, #11360-039], 50 μM 2-mercaptoethanol [Gibco, #31350-010], 50 U/ml Pen:Strep [Gibco #15140-122]) supplemented with 11 mM glucose or 22 mM glucose +/− 5 μM O304. Human islets from non-diabetic donors (Supplementary Table 3) were provided through the JDRF award 31-2008-416 ECIT Islet for Basic Research program in compliance with Swedish law and the Ethical board for human research in Umea (www.epn.se) and were cultured for 48 h in media (CMRL medium [GIBCO #21530-027], 10% fetal bovine serum [GIBCO #10500], 20 U/ml Pen:Strep [Gibco #15140-122] and 1× GlutaMax [Gibco#35050-038]) supplemented with 5.5 mM glucose or 25 mM glucose +/− 5 μM O304.

### INS-1E cell culture

INS-1E cells were obtained from AddexBio Technologies. (AddexBio #C0018009). Cells were maintained RPMI medium 1640 (Gibco #21875- 034), 11.1 mM glucose (Gibco #A24940-01), 10% fetal bovine serum (Gibco #10500-064), 1 mM sodium pyruvate (Gibco #11360-039), 10 mM HEPES (Gibco #15630-056), 0.1% 2-mercaptoethanol (Gibco #31350-010), 50 U/ml Pen;Strep (Gibco #15140- 122) at 37 °C in a humidified incubator with 5% CO_2_. Passages 40–55 were used for all experiments.

### INS-1E western blot and insulin secretion

To mimic non-diabetic/ normoglycemic or diabetic/hyperglycemic conditions INS-1E cells were cultured at 11 mM or 25 mM glucose for 4 days in growth media supplemented with 1% FBS. For studies on the effect of O304 under normo- and hyperglycemic conditions, 5 μM O304 was either added during the entire 4-day culture period (at day 0 and day 2) or during the last 2 h. Next, parallel batches of treated cells were either harvested for protein lysates and western blot or assayed for glucose stimulated insulin secretion (GSIS). GSIS was performed in UB-buffer (125 mM NaCl, 6 mM KCl, 1.3 mM CaCl_2_, 1.2 mM MgCl_2_, 25 mM HEPES (pH 7.3), 2 mM glucose, 0.1% BSA) at 37 °C in normal atmosphere. Briefly, cells were washed and equilibrated for 1 h in UB buffer, transferred to UB buffer containing either 2 or 20 mM glucose for 30 min, and insulin levels in the supernatant was determined by ELISA (Mercodia #10-1249-01). Insulin secretion was normalized to protein content.

### Western blot analysis

Vastus muscles were isolated from non-fasted mice and crushed in a mortar using a pestle and liquid nitrogen. Vastus muscles and INS-1E cell samples were homogenized in ice cold protein lysis buffer (100 mM Tris pH 6.8, 2% SDS), with protease inhibitor cocktail (Roche #04693124001) and phosphatase inhibitor cocktail (Roche #04693124001) and sonicated. The vastus supernatant was collected after 10 min at 14,000 rpm. Protein concentration was measured using the BCA kit (Pierce BCA Protein Assay Kit #23225) after which samples were diluted in Laemmli buffer (BIO-RAD #1610747) and denaturized. 20 µg of vastus and 7 µg of INS-1E cell protein samples were separated on 4–15% CriterionTM TGX Stain-FreeTM Protein Gels (BIO-RAD #5678084) and blotted to low fluorescent PVDF (Trans-Blot Turbo RTA Midi 0.45 µm LF PVDF transfer kit, BIO-RAD #1704275) or Nitrocellulose membrane (Trans-Blot Turbo transfer Pack, Midi, BIO-RAD #1704159), respectively (Supplementary Fig. 9). Primary and secondary antibodies are listed in Supplementary Table 2. Values were normalized to stain-free total protein signal or to a housekeeping gene.

### qRT-PCR

Total RNA from isolated islets was prepared using RNeasy Micro Kit (Qiagen #74004). Total RNA from gastrocnemius muscles and left cardiac ventricles was prepared using RNeasy Fibrous Tissue Mini kit (Qiagen #74704) after first crushing the tissue in liquid nitrogen using a pestle. First strand cDNA synthesis was done using SuperScript III (First-Strand Synthesis SuperMix for qRT-PCR, Invitrogen #11752-250) according to the manufacturer’s instructions. Quantification of mRNA expression levels was performed essentially as previously described^67^. Primers used for qRT-PCR are listed in Supplementary Table 4. Expression of *Tbp* was used for normalization of samples from islets, gastrocnemius muscle and left cardiac ventricle. Normalization by *Tbp* was validated by *Rpl32* in left cardiac ventricle samples and gastrocnemius muscles.

### RNAseq

RNA-seq was performed by The National Genomics Infrastructure (SciLifeLab -Science for Life Laboratory, SNP&SEQ Technology Platform in Uppsala). RNA-seq libraries were prepared from 150 ng total RNA using the Illumina TruSeq stranded mRNA Library Kit (Cat# 20020595) followed by 100 bp paired-end sequencing on a NovaSeq 6000 Illumina Sequencer. RNA-seq reads were processed using resources from Swedish National Infrastructure for Computing (SNIC) at UPPMAX. Fastq files with 100-nt paired-end sequenced reads were quality-checked with *FastQC* (https://www.bioinformatics.babraham.ac.uk/projects/fastqc/), aligned to the mouse or human genome (GRCm39 or GRCh38, version 103, ensembl.org) using *STAR* (default options)^68^. Fragment-gene hits were counted by *featureCounts* (options: -p -t gene -g gene_id -s 2)^69^. Subsequent analysis was performed in R using packages *DESeq2* and clusterProfiler^70,71^. Normalization and differential expression analysis were performed using *DESeq2*^71^ with batch correction (referring to islet preparation batch and human donor respectively), independent filtering (alpha = 0.05) and False Discovery Rate (FDR) < 0.05. Genes with baseMean expression >filterThreshold (in the DEseqResults metadata independent filtering) were considered to be expressed and to constitute the gene background for subsequent ORA and GSEA analysis using *clusterProfiler*^70^ with a pvalueCutoff = 0.05 and a qvalueCutoff = 0.2 for overrepresented gene categories.

### Statistics and reproducibility

Quantification of cell numbers from pancreatic sections was performed using Image-J software (version1.49 m). The experimental design was considered to consist of a non-diabetic control group (BKS or Control (i.e., vehicle injected F1 mice), a diabetic control (db/db mice or STZ mice, i.e., STZ injected F1 mice) and O304-treated group(s) (db/db mice or STZ mice kept on a diet formulated with O304). First, the validity of the diabetic models was tested by comparing the control and diabetic groups. Second, the effect of O304 diet was evaluated by comparing the O304-treated group(s) to the diabetic group. The parametric nature and heteroscedasticity of the data were evaluated by Shapiro–Wilk test and Leven’s test and used to select the appropriate statistical test. For two-group comparisons, heteroscedastic *t* test or Wilcoxon test were used together with holm correction for multiple testing. For multiple groups, ANOVA analysis (one-way ANOVA, Welch’s ANOVA, Kruskal–Wallis Test, One-way ANOVA with repeated testing), were used followed by the appropriate post hoc analysis (see figure legends). *P* < 0.05 was considered as statistically significant. Data analysis, statistical analysis and visualization were performed in R using the *tidyverse*, *rstatix* and *ggpubr* packages^72–74^. Number of animals or independent experiments (*n*) are indicated in the figure legend. Data are presented as mean ± standard error of the mean (SEM) to visualize reproducibility between replicate experiments.

### Reporting summary

Further information on research design is available in the Nature Portfolio Reporting Summary linked to this article.

### Supplementary information

Supplementary information

Description of Additional Supplementary Files

Supplementary Data 1

Reporting Summary

## Data Availability

All data generated and/or analyzed during this study are either included in this article (and its Supplementary Information and Supplementary Data 1) or are available from the corresponding author on reasonable request. All generated datasets for RNA-seq are available through the Gene Expression Omnibus repository under the accession number GSE240298.

## References

[1] DeFronzo RA (1981). The effect of insulin on the disposal of intravenous glucose. Results from indirect calorimetry and hepatic and femoral venous catheterization. Diabetes.

[2] DeFronzo RA, Gunnarsson R, Bjorkman O, Olsson M, Wahren J (1985). Effects of insulin on peripheral and splanchnic glucose metabolism in noninsulin-dependent (type II) diabetes mellitus. J. Clin. Investig..

[3] Best JD (1996). Role of glucose effectiveness in the determination of glucose tolerance. Diabetes Care.

[4] Ahren B, Pacini G (2021). Glucose effectiveness: lessons from studies on insulin-independent glucose clearance in mice. J. Diabetes Investig..

[5] Alford FP, Henriksen JE, Rantzau C, Beck-Nielsen H (2018). Glucose effectiveness is a critical pathogenic factor leading to glucose intolerance and type 2 diabetes: an ignored hypothesis. Diabetes Metab. Res. Rev..

[6] Bruce CR (2021). Translating glucose tolerance data from mice to humans: insights from stable isotope labelled glucose tolerance tests. Mol. Metab..

[7] Galante P (1995). Acute hyperglycemia provides an insulin-independent inducer for GLUT4 translocation in C2C12 myotubes and rat skeletal muscle. Diabetes.

[8] Mevorach M (1998). Regulation of endogenous glucose production by glucose per se is impaired in type 2 diabetes mellitus. J. Clin. Investig..

[9] Del Prato S (1997). Studies on the mass action effect of glucose in NIDDM and IDDM: evidence for glucose resistance. Diabetologia.

[10] Steneberg P (2018). PAN-AMPK activator O304 improves glucose homeostasis and microvascular perfusion in mice and type 2 diabetes patients. JCI Insight.

[11] Cokorinos EC (2017). Activation of skeletal muscle AMPK promotes glucose disposal and glucose lowering in non-human primates and mice. Cell Metab..

[12] Myers RW (2017). Systemic pan-AMPK activator MK-8722 improves glucose homeostasis but induces cardiac hypertrophy. Science.

[13] Fujitani J (1998). Intravenous glucose tolerance test-derived glucose effectiveness in strength-trained humans. Metabolism.

[14] Tokuyama K (1993). Intravenous glucose tolerance test-derived glucose effectiveness in physically trained humans. Am. J. Physiol..

[15] Esquejo RM (2022). AMPK activation is sufficient to increase skeletal muscle glucose uptake and glycogen synthesis but is not required for contraction-mediated increases in glucose metabolism. Heliyon.

[16] Ericsson M, Steneberg P, Nyren R, Edlund H (2021). AMPK activator O304 improves metabolic and cardiac function, and exercise capacity in aged mice. Commun. Biol..

[17] van Raalte DH, Verchere CB (2017). Improving glycaemic control in type 2 diabetes: stimulate insulin secretion or provide beta-cell rest?. Diabetes Obes. Metab..

[18] Erion K, Corkey BE (2018). Beta-cell failure or beta-cell abuse?. Front. Endocrinol..

[19] Alarcon C (2016). Pancreatic beta-cell adaptive plasticity in obesity increases insulin production but adversely affects secretory function. Diabetes.

[20] Boland BB, Rhodes CJ, Grimsby JS (2017). The dynamic plasticity of insulin production in beta-cells. Mol. Metab..

[21] Nolan CJ, Delghingaro-Augusto V (2016). Reversibility of defects in proinsulin processing and islet beta-cell failure in obesity-related type 2 diabetes. Diabetes.

[22] Glaser B (1988). Improved beta-cell function after intensive insulin treatment in severe non-insulin-dependent diabetes. Acta Endocrinol..

[23] Qvigstad E, Kollind M, Grill V (2004). Nine weeks of bedtime diazoxide is well tolerated and improves beta-cell function in subjects with Type 2 diabetes. Diabet. Med..

[24] Lim EL (2011). Reversal of type 2 diabetes: normalisation of beta cell function in association with decreased pancreas and liver triacylglycerol. Diabetologia.

[25] Taylor R (2018). Remission of human type 2 diabetes requires decrease in liver and pancreas fat content but is dependent upon capacity for beta cell recovery. Cell Metab..

[26] Camastra S (2013). Long-term effects of bariatric surgery on meal disposal and beta-cell function in diabetic and nondiabetic patients. Diabetes.

[27] Parikh H (2007). TXNIP regulates peripheral glucose metabolism in humans. PLoS Med..

[28] Wu N (2013). AMPK-dependent degradation of TXNIP upon energy stress leads to enhanced glucose uptake via GLUT1. Mol. Cell.

[29] Waldhart AN (2017). Phosphorylation of TXNIP by AKT mediates acute influx of glucose in response to insulin. Cell Rep..

[30] Mukai N, Nakayama Y, Abdali SA, Yoshioka J (2021). Cardiomyocyte-specific Txnip C247S mutation improves left ventricular functional reserve in streptozotocin-induced diabetic mice. Am. J. Physiol. Heart Circ. Physiol..

[31] Shalev A (2014). Minireview: thioredoxin-interacting protein: regulation and function in the pancreatic beta-cell. Mol. Endocrinol..

[32] Kawaguchi T, Osatomi K, Yamashita H, Kabashima T, Uyeda K (2002). Mechanism for fatty acid “sparing” effect on glucose-induced transcription: regulation of carbohydrate-responsive element-binding protein by AMP-activated protein kinase. J. Biol. Chem..

[33] Jeon JH (2021). Loss of metabolic flexibility as a result of overexpression of pyruvate dehydrogenase kinases in muscle, liver and the immune system: therapeutic targets in metabolic diseases. J Diabetes Investig..

[34] Zhang S, Hulver MW, McMillan RP, Cline MA, Gilbert ER (2014). The pivotal role of pyruvate dehydrogenase kinases in metabolic flexibility. Nutr. Metab..

[35] Samec S, Seydoux J, Dulloo AG (1998). Role of UCP homologues in skeletal muscles and brown adipose tissue: mediators of thermogenesis or regulators of lipids as fuel substrate?. FASEB J..

[36] Rohm M (2018). Cardiac dysfunction and metabolic inflexibility in a mouse model of diabetes without dyslipidemia. Diabetes.

[37] Puff R (2011). Reduced proliferation and a high apoptotic frequency of pancreatic beta cells contribute to genetically-determined diabetes susceptibility of db/db BKS mice. Horm. Metab. Res..

[38] Shimamura M, Karasawa H, Sakakibara S, Shinagawa A (2010). Raldh3 expression in diabetic islets reciprocally regulates secretion of insulin and glucagon from pancreatic islets. Biochem. Biophys. Res. Commun..

[39] Flisher MF, Shin D, Huising MO (2022). Urocortin3: local inducer of somatostatin release and bellwether of beta cell maturity. Peptides.

[40] Liberzon A (2015). The Molecular Signatures Database (MSigDB) hallmark gene set collection. Cell Syst..

[41] Jaafar R (2019). mTORC1 to AMPK switching underlies beta-cell metabolic plasticity during maturation and diabetes. J. Clin. Investig..

[42] Haythorne E (2022). Altered glycolysis triggers impaired mitochondrial metabolism and mTORC1 activation in diabetic beta-cells. Nat. Commun..

[43] Taylor SI, Yazdi ZS, Beitelshees AL (2021). Pharmacological treatment of hyperglycemia in type 2 diabetes. J. Clin. Investig..

[44] Jain C, Ansarullah, Bilekova S, Lickert H (2022). Targeting pancreatic beta cells for diabetes treatment. Nat. Metab..

[45] Dube S, Errazuriz-Cruzat I, Basu A, Basu R (2015). The forgotten role of glucose effectiveness in the regulation of glucose tolerance. Curr. Diab. Rep..

[46] Ebeling P, Koistinen HA, Koivisto VA (1998). Insulin-independent glucose transport regulates insulin sensitivity. FEBS Lett..

[47] Cartee GD, Wojtaszewski JF (2007). Role of Akt substrate of 160 kDa in insulin-stimulated and contraction-stimulated glucose transport. Appl. Physiol. Nutr. Metab..

[48] Richter EA, Hargreaves M (2013). Exercise, GLUT4, and skeletal muscle glucose uptake. Physiol. Rev..

[49] Dimitrakoudis D, Ramlal T, Rastogi S, Vranic M, Klip A (1992). Glycaemia regulates the glucose transporter number in the plasma membrane of rat skeletal muscle. Biochem. J..

[50] Randle PJ (1966). Interactions of metabolism and the physiological role of insulin. Recent Prog. Horm. Res..

[51] Capaldo B, Santoro D, Riccardi G, Perrotti N, Sacca L (1986). Direct evidence for a stimulatory effect of hyperglycemia per se on peripheral glucose disposal in type II diabetes. J. Clin. Investig..

[52] Vaag A, Damsbo P, Hother-Nielsen O, Beck-Nielsen H (1992). Hyperglycaemia compensates for the defects in insulin-mediated glucose metabolism and in the activation of glycogen synthase in the skeletal muscle of patients with type 2 (non-insulin-dependent) diabetes mellitus. Diabetologia.

[53] Mellor, K. M. et al. Protective role of the Atg8 homologue Gabarapl1 in regulating cardiomyocyte glycophagy in diabetic heart disease. Preprint at *bioRxiv*10.1101/2021.06.21.449174 (2021).

[54] Kjobsted R (2019). AMPK and TBC1D1 regulate muscle glucose uptake after, but not during, exercise and contraction. Diabetes.

[55] Axelrod CL (2020). BAM15-mediated mitochondrial uncoupling protects against obesity and improves glycemic control. EMBO Mol. Med..

[56] Malik N (2023). Induction of lysosomal and mitochondrial biogenesis by AMPK phosphorylation of FNIP1. Science.

[57] Brahma MK, Pepin ME, Wende AR (2017). My sweetheart is broken: role of glucose in diabetic cardiomyopathy. Diabetes Metab. J..

[58] Chen YC, Taylor AJ, Verchere CB (2018). Islet prohormone processing in health and disease. Diabetes Obes. Metab..

[59] Rhodes CJ, Alarcon C (1994). What beta-cell defect could lead to hyperproinsulinemia in NIDDM? Some clues from recent advances made in understanding the proinsulin-processing mechanism. Diabetes.

[60] Lopez-Perez A (2021). Pan-AMPK activator O304 prevents gene expression changes and remobilisation of histone marks in islets of diet-induced obese mice. Sci. Rep..

[61] Bertholet AM (2022). Mitochondrial uncouplers induce proton leak by activating AAC and UCP1. Nature.

[62] Cochran BJ (2017). Determining Glucose Metabolism Kinetics Using 18F-FDG Micro-PET/CT. J. Vis. Exp..

[63] Patlak CS, Blasberg RG, Fenstermacher JD (1983). Graphical evaluation of blood-to-brain transfer constants from multiple-time uptake data. J. Cereb. Blood Flow Metab..

[64] Williams SP, Flores-Mercado JE, Baudy AR, Port RE, Bengtsson T (2012). The power of FDG-PET to detect treatment effects is increased by glucose correction using a Michaelis constant. EJNMMI Res..

[65] Norlin S, Parekh VS, Naredi P, Edlund H (2016). Asna1/TRC40 controls beta-cell function and endoplasmic reticulum homeostasis by ensuring retrograde transport. Diabetes.

[66] Ahren B (1997). Dissociated insulinotropic sensitivity to glucose and carbachol in high-fat diet-induced insulin resistance in C57BL/6J mice. Metabolism.

[67] Steneberg P, Rubins N, Bartoov-Shifman R, Walker MD, Edlund H (2005). The FFA receptor GPR40 links hyperinsulinemia, hepatic steatosis, and impaired glucose homeostasis in mouse. Cell Metab..

[68] Dobin A (2013). STAR: ultrafast universal RNA-seq aligner. Bioinformatics.

[69] Liao Y, Smyth GK, Shi W (2014). featureCounts: an efficient general purpose program for assigning sequence reads to genomic features. Bioinformatics.

[70] Wu T (2021). clusterProfiler 4.0: a universal enrichment tool for interpreting omics data. Innovation.

[71] Love MI, Huber W, Anders S (2014). Moderated estimation of fold change and dispersion for RNA-seq data with DESeq2. Genome Biol..

[72] ggpubr: ‘ggplot2’ Based Publication Ready Plots. R package version 0.4.0. https://CRAN.R-project.org/package=ggpubr (2020).

[73] rstatix: Pipe-Friendly Framework for Basic Statistical Tests. R package version 0.7.0. https://CRAN.R-project.org/package=rstatix (2021).

[74] Hadley Wickham, M. A. et al. Welcome to the Tidyverse. *J. Open Sour. Softw.* **4**, 10.21105/joss.01686 (2019).

