# Supplementary material for: O304 ameliorates hyperglycemia in mice by dually promoting muscle glucose effectiveness and preserving β-cell function
Source: Commun Biol. 2023 Aug 25;6:877. doi: 10.1038/s42003-023-05255-6 (PMC10457357; doi:10.1038/s42003-023-05255-6)
Supplement: Supplementary file 1 — Supplementary information [file 42003_2023_5255_MOESM1_ESM.pdf]

Supplementary information

O304 ameliorates hyperglycemia in mice by dually promoting muscle glucose effectiveness and preserving β-cell function

Stefan Norlin[1], Jan Axelsson[2], Madelene Ericsson[1], and Helena Edlund[1*]
[1]Umeå Centre for Molecular Medicine

Umeå University,
SE-901 87 Umeå, Sweden
[2]Department of Radiation Sciences, Radiation Physics
Umeå University,
SE-901 87 Umeå, Sweden

Supplementary figure 1. Immunostaining and non-fasted glucose levels in STZ mice

Supplementary figure 2. SUV profiles during FDG-PET scanning of control and STZ mice

Supplementary figure 3. Quantification of pAMPK/AMPK, pACC/ACC and TXNIP in skeletal muscle of control and STZ mice

Supplementary figure 4. O304 treatment does not increase serum levels of lactate in STZ and db/db mice

Supplementary figure 5. Quantification of PP^+ and Som^+ cell fraction and total pancreatic proinsulin content in BKS and db/db mice.

Supplementary figure 6. RNA seq data from analyses of *ex vivo* cultured mouse and human islets

Supplementary figure 7. Representative immunoblot of AMPK and mTORC1 signalling components in INS-1E cells

Supplementary figure 8. Schematic model summarizing the effects of O304

Supplementary figure 9. Uncropped western blots

Supplementary table 1. Echocardiographic measurements

Supplementary table 2. List of antibodies

Supplementary table 3. Human islet donor information

Supplementary table 4. List of primers for qRT-PCR

Supplementary figure 1.

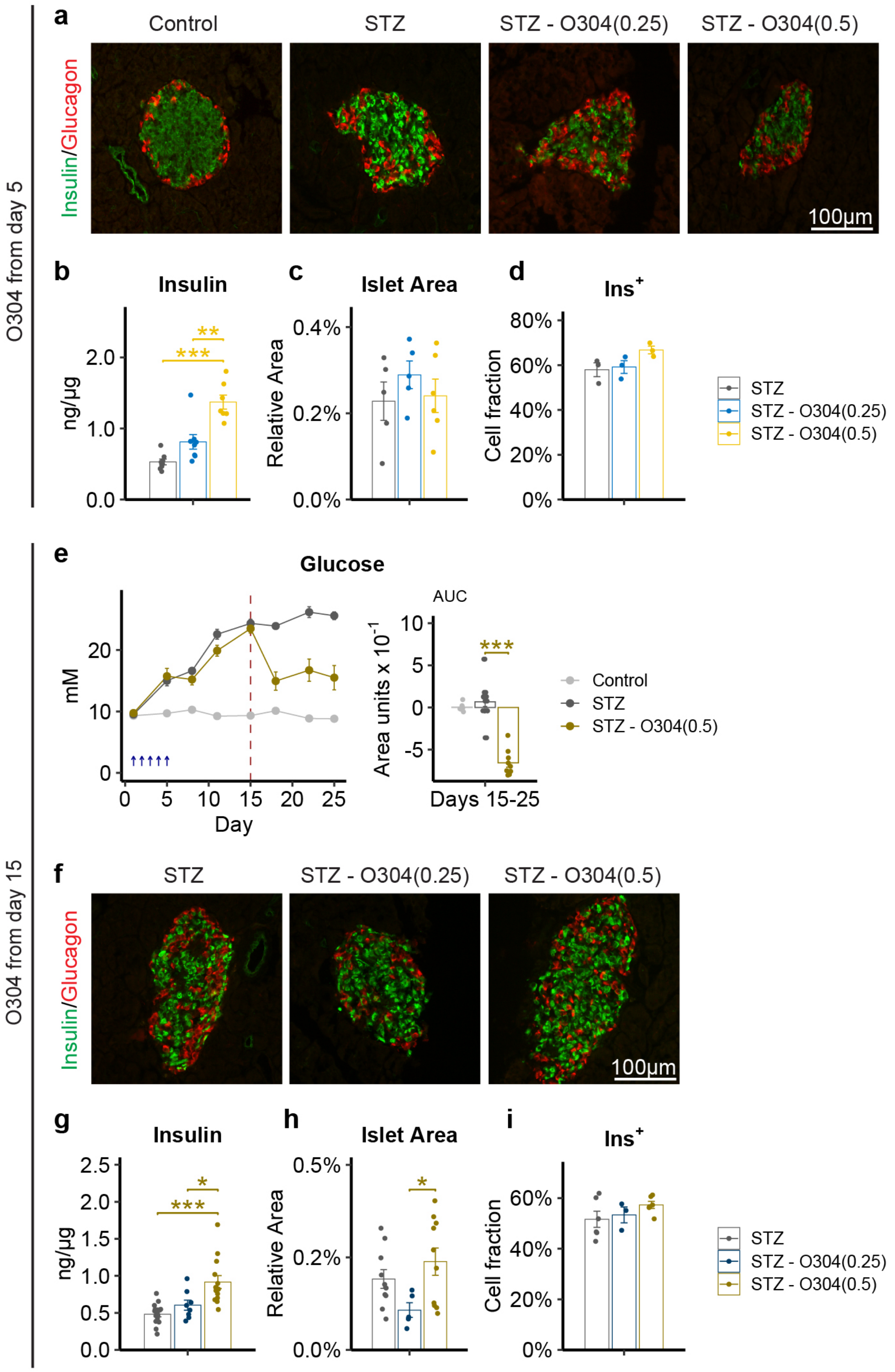

Supplementary figure 1. Immunohistochemical and metabolic analyses of control and STZ mice. a Representative insulin (green) and glucagon (red) double immunostaining of

pancreases from control and STZ mice untreated or treated with 0.25 and 0.5 mg/g O304 from day 5 (n=5 for all groups). **b** Total pancreatic insulin content in STZ mice untreated or treated with 0.25 and 0.5 mg/g O304, respectively, from day 5 (n=7-8/group). **c**,**d** Islet cell area (**c**) and Insulin (Ins$^+$) (**d**) cell fraction in STZ mice untreated (n=3-5) or treated with 0.25 (n=3-5) and 0.5 (n=3-6) mg/g O304, respectively, from day 5. **e** Non-fasted glucose levels with area under the curve (AUC) in control (n=6) and STZ mice untreated (n=9) or treated with 0.5 (n=9) mg/g O304, respectively, from day 15. **f** Representative insulin and glucagon double immunostaining of pancreases from STZ mice untreated or treated with 0.25 and 0.5 mg/g O304, respectively, from day 15 (n=5 for all groups). **g** Total pancreatic insulin content in STZ mice untreated or treated with 0.25 and 0.5 mg/g O304, respectively, from day 15 (n=8-16/group). **h,i** Islet cell area (**h**) and Ins$^+$ (**i**) cell fraction in STZ mice untreated (n=6-10) or treated with 0.25 (n=3-5) and 0.5 (n=6-10) mg/g O304 from day 15. Data are presented as mean ± SEM. *P<0.05, **P<0.01, ***P<0.001, by Welch's ANOVA followed by Games-Howell *post hoc* test (**b-c**, **g-h**) or one-way ANOVA followed by Tukey's *post hoc* test (**d**, **i**) or Student's t-test (**f**).

Supplementary figure 2.

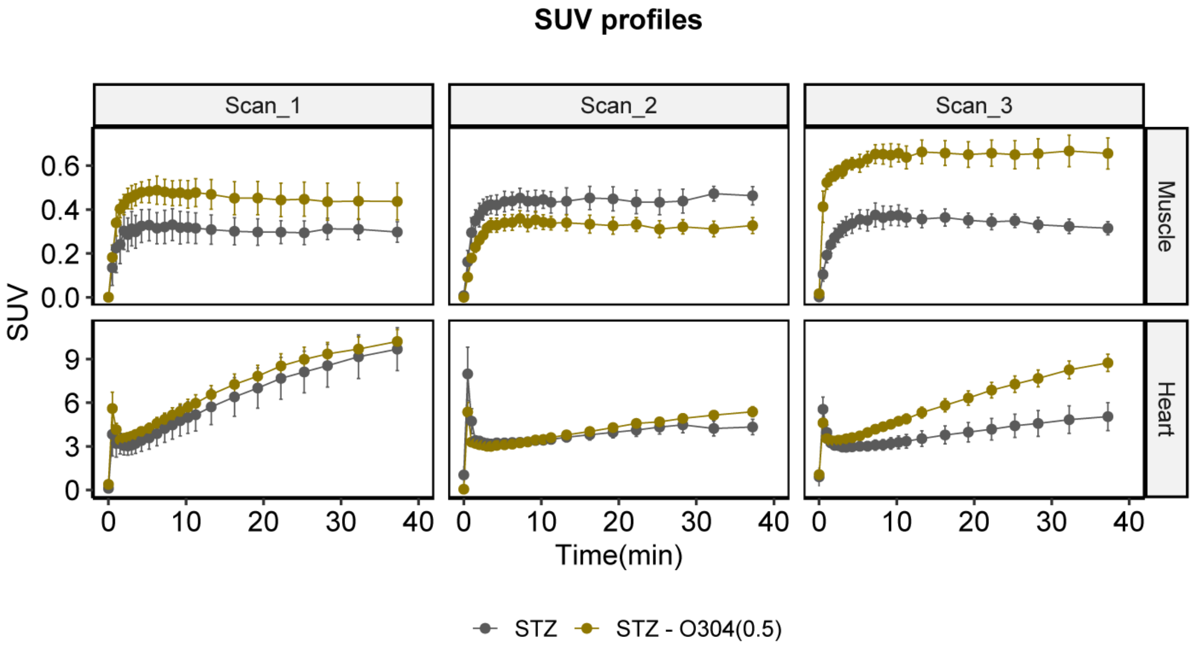

Supplementary figure 2. Standardized Uptake Values (SUV) during FDG-PET scanning. SUV profiles of gastrocnemius muscle and heart during a 40-minute dynamic FDG-PET scan at baseline (Scan 1), 9-10 days after the last STZ injection (Scan 2), and after 1 week of treatment (Scan 3) with 0.5mg/g O304 (n=5) or no treatment (n=5). Data are presented as mean $\pm$ SEM.

Supplementary figure 3.

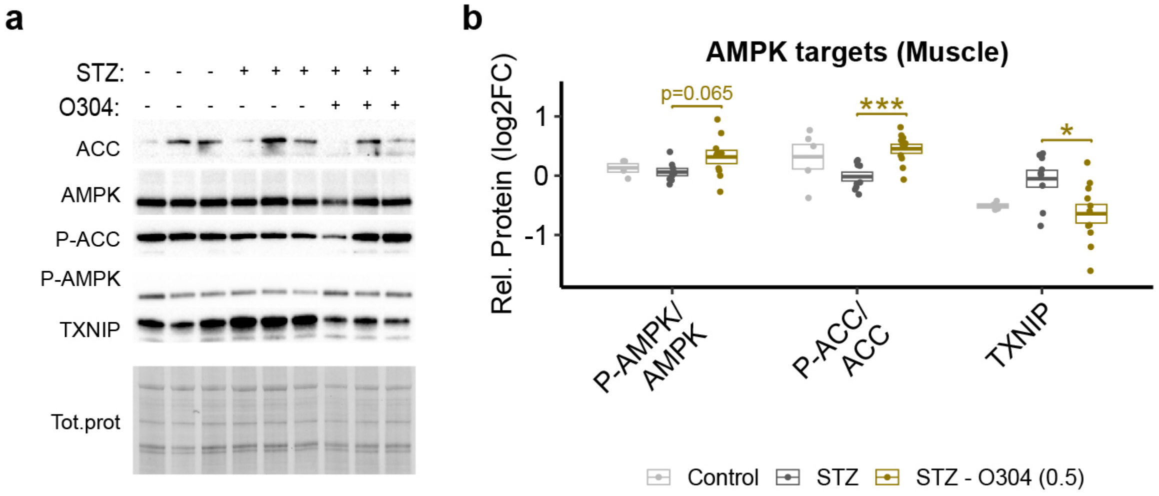

Supplementary figure 3. Representative immunoblots **(a)** and quantification **(b)** of protein levels by western blot analyses of p-T172 AMPK, p-S79 ACC, and TXNIP protein levels in extracts from vastus muscle in control (n= 5) and STZ mice untreated (n=9) or treated with 0.5 mg/g O304 (n=9) from day 15.

Supplementary figure 4.

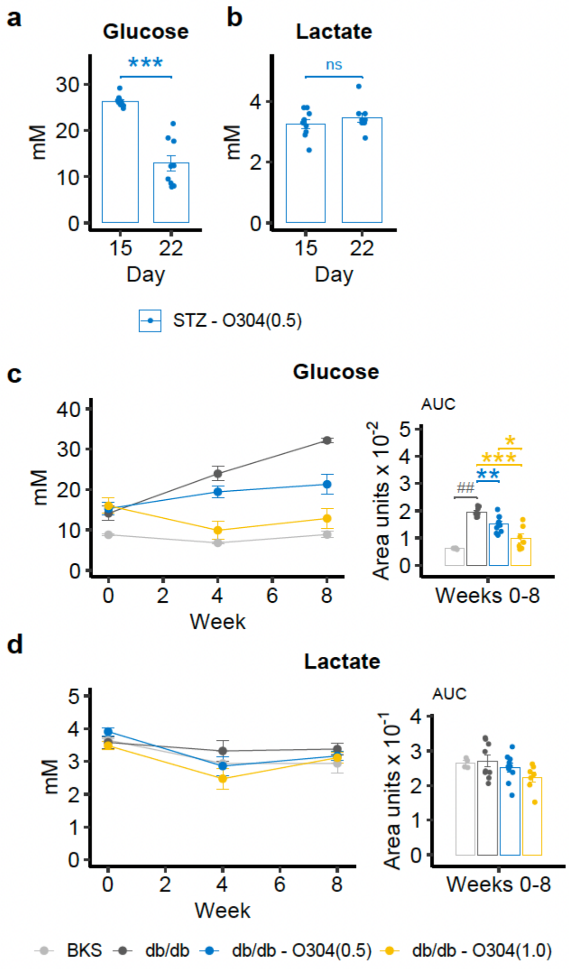

Supplementary figure 4. O304 treatment does not increase serum levels of lactate in STZ and db/db mice. a,b Fasted glucose (**a**) and lactate (**b**) levels in STZ mice at day 15 (i.e. before treatment) and at day 22 after 1 week treatment with 0.5 mg/g O304 (n=9/group). **c,d** Fasted glucose (**c**) and lactate (**d**) levels with area under the curve (AUC) in BKS and db/db mice untreated or treated with 0.5 and 1.0 mg/g O304, respectively (n=7-9/group). Data are presented as mean $\pm$ SEM. Statistical significance between timepoints in (a) was determined by Student's t-test **P*<0.05, and between untreated and O304 treated db/db mice was determined by Welch's

ANOVA followed by Games-Howell *post hoc* test (*P<0.05, **P<0.01, ***P<0.001), and between BKS and db/db mice was determined by Wilcoxon test in (c,d) ($^{\#\#}P$<0.01)

Supplementary figure 5.

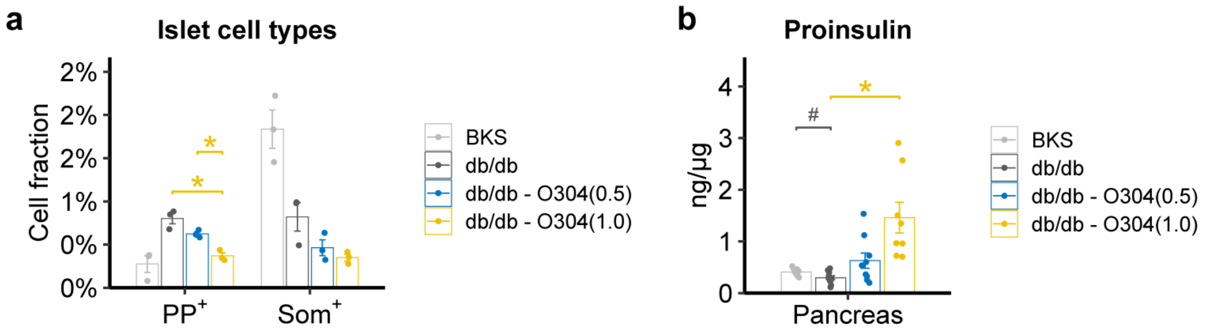

Supplementary figure 5. PP$^+$ and Som$^+$ cell fraction and total pancreatic proinsulin content in BKS and db/db mice. a Pancreatic polypeptide (PP$^+$) and somatostatin (Som$^+$) positive cell fraction in 15 w old BKS and db/db mice untreated or treated with 0.5 and 1.0 mg/g O304 (n= 3 for each group). **b** Total pancreatic proinsulin content in 15w old BKS (n=8) and db/db mice untreated or treated with 0.5 and 1.0 mg/g O304, respectively, (n=8-10/group). Data are presented as mean ± SEM**.** *P<0.05, by Welch's ANOVA followed by Games-Howell *post hoc* test, between untreated and O304 treated db/db mice. $^{\#}P$<0.05, by Wilcoxon test, between BKS and db/db mice

Supplementary figure 6.

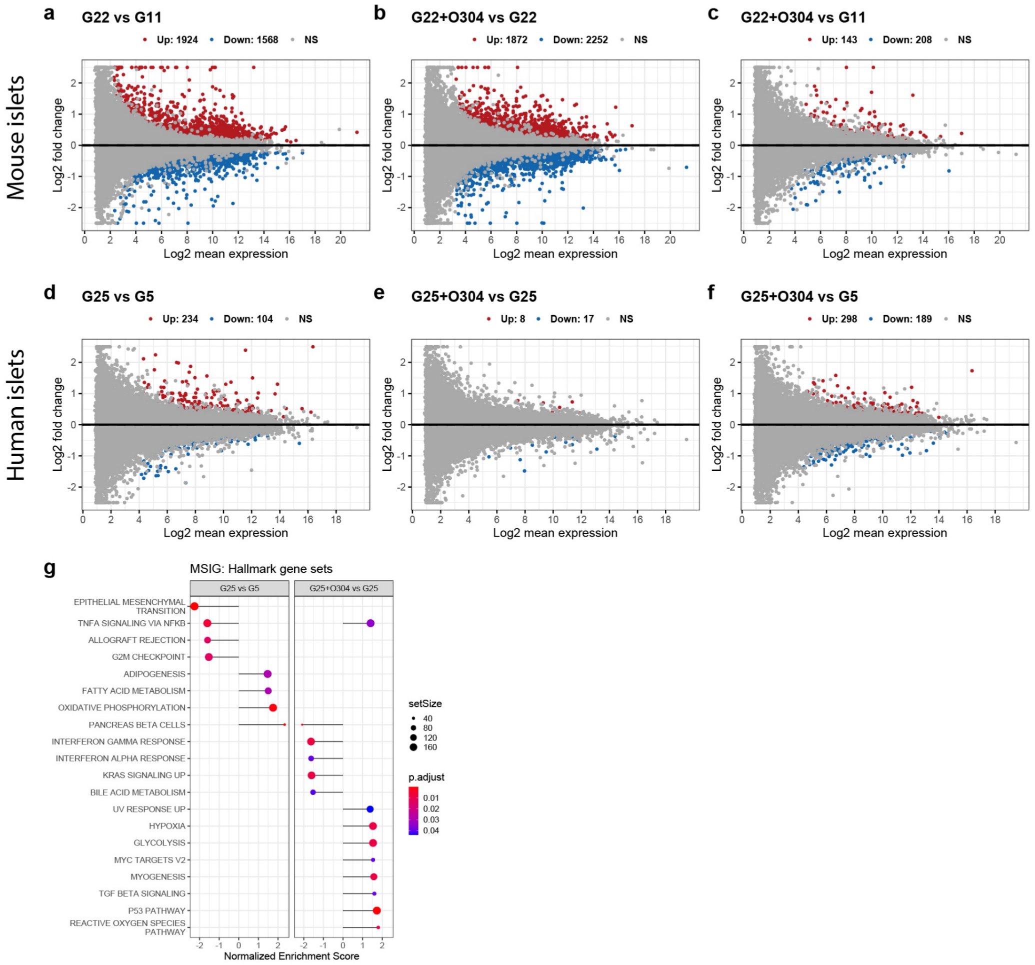

Supplementary figure 6. O304 effects on gene expression signatures in *ex vivo* cultured mouse and human islets.

a-c MA plots showing differentially expressed genes between mouse islets cultured at 22mM (G22) vs 11mM (G11) glucose (**a**), 22mM glucose +5µM O304 (G22+O304) vs 22mM glucose (**b**) and 22mM glucose + 5µM O304 vs 11mM glucose (**c**). **d-f** MA plots showing differentially expressed genes between human islets cultured at 25mM (G25) vs 5.5mM (G5) glucose (**d**), 25mM glucose + 5µM O304 (G25+O304) vs 25mM glucose (**e**), and 25mM glucose + 5µM O304 vs 5.5mM glucose (**f**). **g** Overrepresentation analysis (ORA) of Molecular signature Hallmark gene sets in human islets cultured at 22mM vs 11mM glucose and 22mM glucose + 5µM O304 vs 22mM glucose.

Supplementary figure 7.

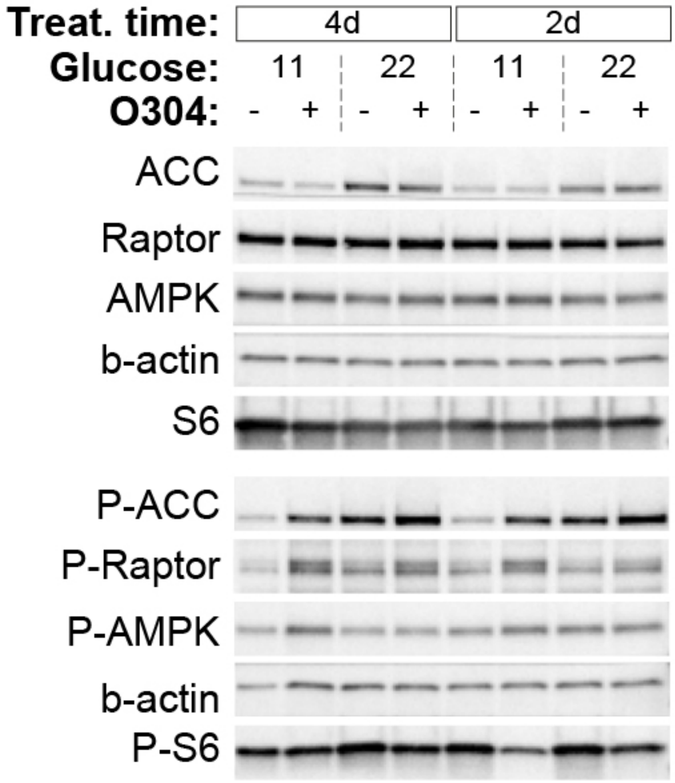

Supplementary figure 7. Representative immunoblot of INS-1E cells cultured under 11mM or 25mM glucose for 4 d, untreated and treated with 5μM O304 for 4days (4d) or 2 hours (2h).

Supplementary figure 8.

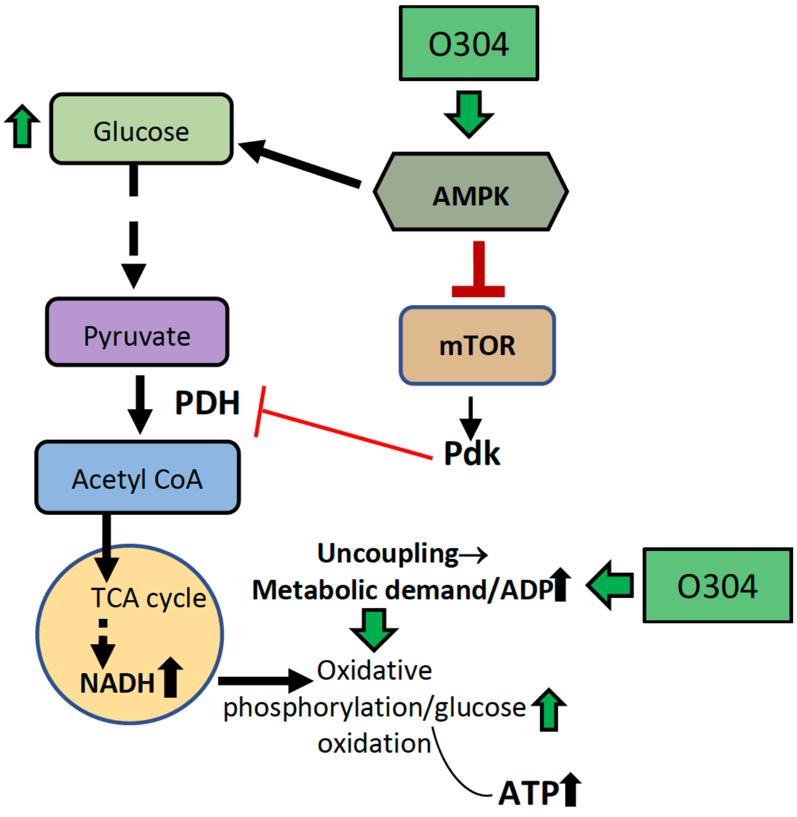

Supplementary figure 8. Schematic model for anti-diabetic effects of O304. As a dual AMPK activator and mitochondrial uncoupler O304 stimulates glucose uptake and utilization in muscle and averts the glucotoxicity effects of hyperglycemia on β-cell function, in part via reduction of mTORC1 signalling and thus Pdk expression.

Supplementary figure 9a. Uncropped and un-edited images of blots of AMPK, pAMPK, ACC, pACC, and TXNIP shown in Suppl. fig. 3a

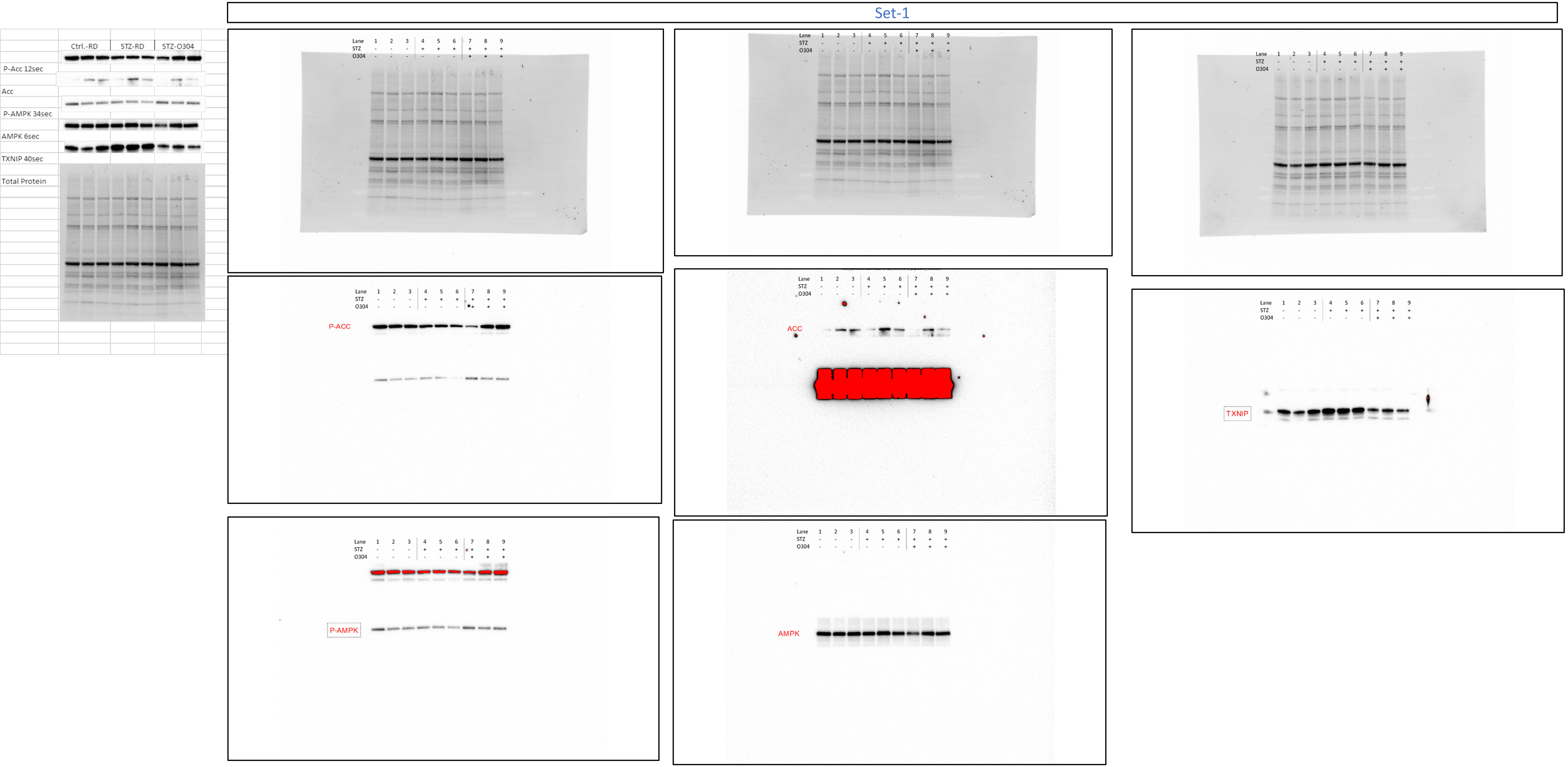

Uncropped and un-edited images of blots shown in

Supplementary figure 9b. Uncropped and un-edited images of AMPK, pAMPK, ACC, pACC, and TXNIP blots shown in Suppl. fig. 3a

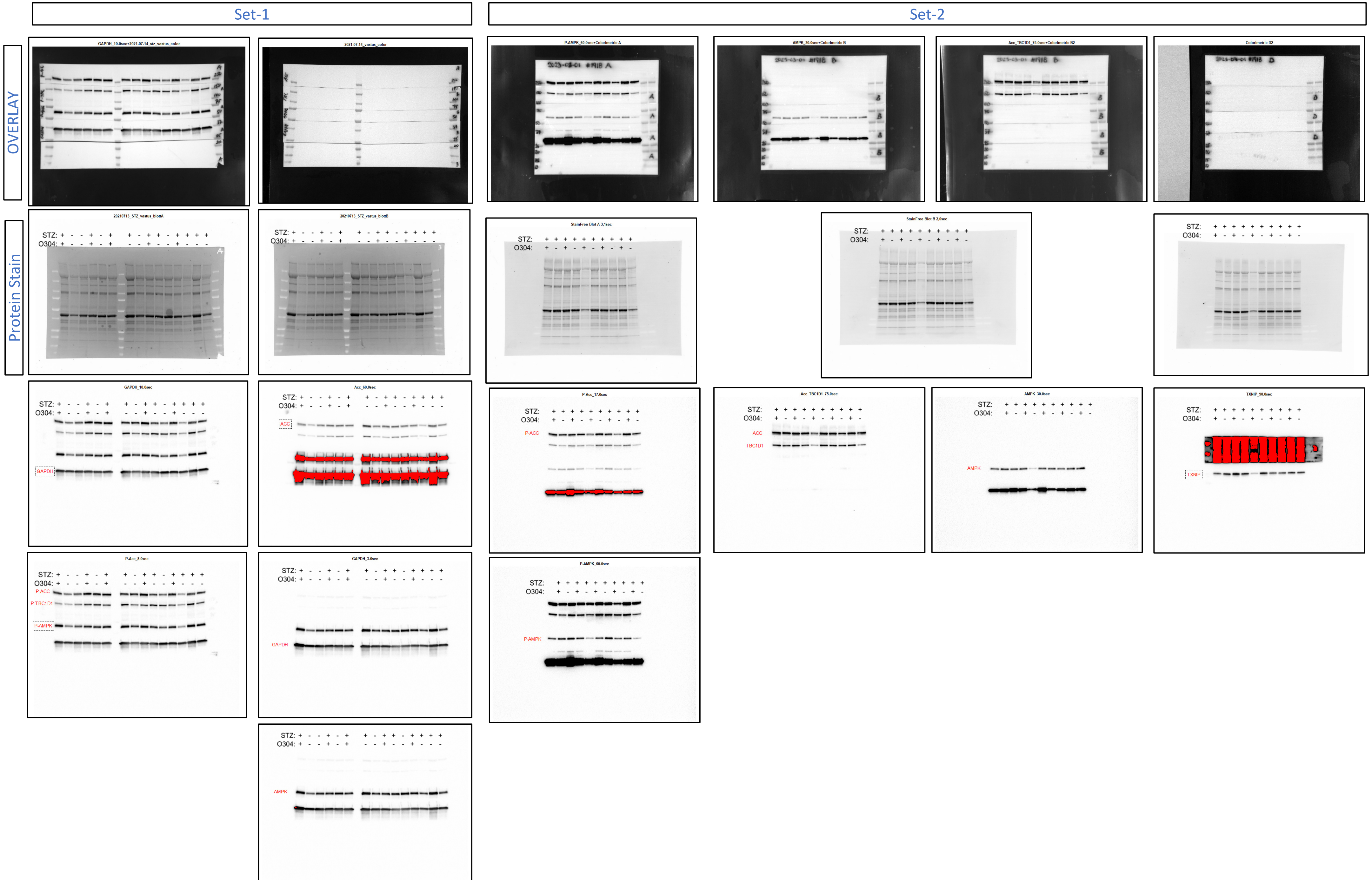

Supplementary figure 9c. Uncropped and un-edited images of AMPK, pAMPK, ACC, pACC, Raptor, pRaptor, S6, and pS6 blots shown in Suppl. fig. 7.

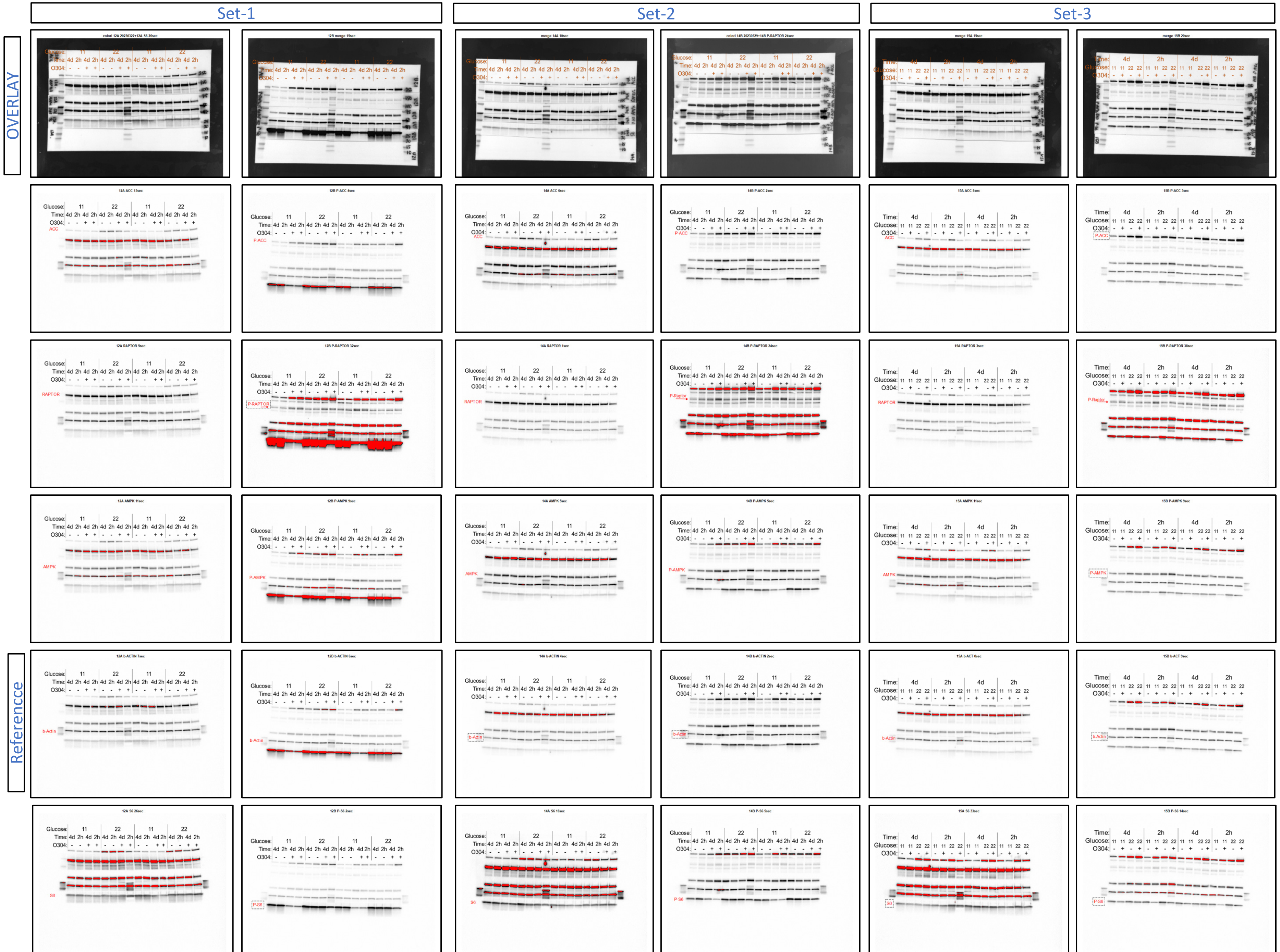

Supplementary figure 9d. Uncropped and un-edited images of AMPK, pAMPK, ACC, pACC, Raptor, pRaptor, S6, and pS6 blots shown in Suppl. fig. 7.

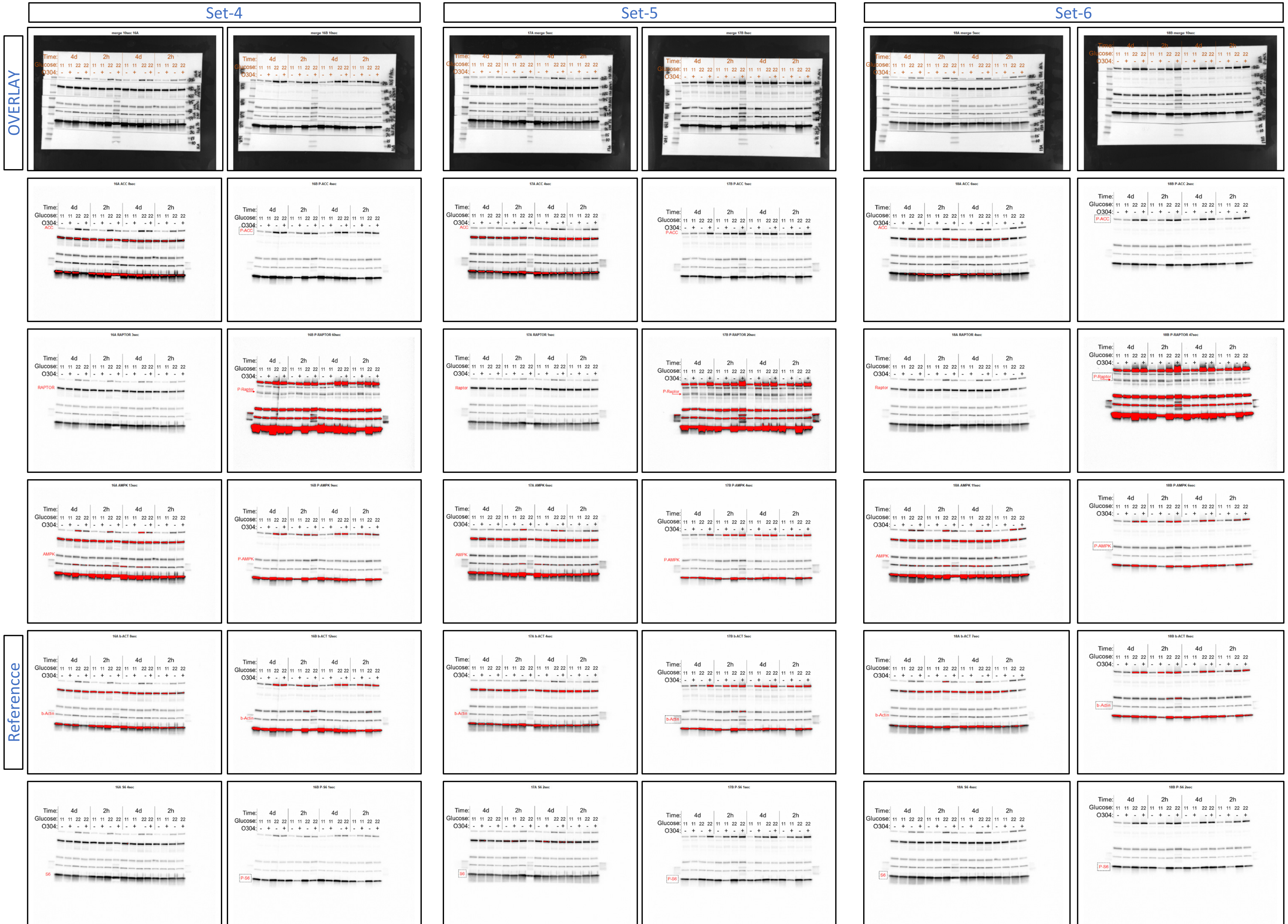

Supplementary table 1.

Echocardiographic measurements

	RD	O304	RD	O304	RD	O304
	Scan 1		Scan 2		Scan 3	
n	9	9	9	9	9	9
PLAX-B-mode						
HR (bpm)	429± 46	411 ± 35	396 ± 55	387 ± 37	421 ± 66	445 ± 52
SV (µL)	22.3 ± 1.15	22.6 ± 0.64	20.2 ± 1.16#	20.8 ± 1.91#	19.5 ± 1.76##	22.8 ± 1.53***¤
CO (mL/min)	9.5 ± 1.18	9.3 ± 0.87	8.0 ± 1.25	8.1 ± 1.10	8.3 ± 1.66	10.1 ± 1.17*
EDV (µL)	57.6 ± 6.36	56.2 ± 8.52	53.3 ± 6.56	49.9 ± 7.51	51.8 ± 6.48	56.6 ± 5.26
ESV (µL)	35.3 ± 6.18	33.6 ± 8.58	33.0 ± 6.20	29.1 ± 6.59	32.3 ± 5.70	33.8 ± 4.69
PLAX M-mode						
AWd (mm)	0.45 ± 0.03	0.47 ± 0.02	0.48 ± 0.02	0.50 ± 0.01##**	0.50 ± 0.04#	0.46 ± 0.02*¤¤¤
AWs (mm)	0.56 ± 0.02	0.56 ± 0.03	0.57 ± 0.03	0.59 ± 0.03	0.59 ± 0.04	0.53 ± 0.03**
LVIDd (mm)	4.44 ± 0.29	4.46 ± 0.23	4.45 ± 0.34	4.36 ± 0.27	4.29 ± 0.30	4.44 ± 0.20
LVIDs (mm)	3.41 ± 0.30	3.38 ± 0.28	3.54 ± 0.29	3.39 ± 0.36	3.35 ± 0.29	3.38 ± 0.28
PWd (mm)	0.67 ± 0.30	0.68 ± 0.13	0.65 ± 0.08	0.64 ± 0.08	0.68 ± 0.06	0.62 ± 0.05
PWs (mm)	0.83 ± 0.07	0.80 ± 0.08	0.77 ± 0.10	0.78 ± 0.08	0.82 ± 0.07	0.81 ± 0.10
EF (%)	46.7 ± 5.73	48.2 ± 4.90	42.0 ± 2.90	45.2 ± 6.68	44.3 ± 6.58	47.9 ± 5.40
FS (%)	23.3 ± 3.41	24.2 ± 2.97	20.5 ± 1.66	22.4 ± 3.80	21.9 ± 3.94	24.0 ± 3.20
E/A	1.97 ± 0.57	1.95 ± 0.46	1.88 ± 0.45	2.02 ± 0.77	1.68 ± 0.28	2.97 ± 0.77##**¤
E peak velocity (mm/s)	622 ± 103	650 ± 69	565 ± 84	589 ± 30	494 ± 125	707 ± 78**¤¤¤
A peak velocity (mm/s)	340 ± 108	348 ± 85	309 ± 56	349 ± 141	310 ± 122	247 ± 62
Decel time (ms)	22.4 ± 4.26	20.8 ± 4.76	21.0 ± 4.15	20.3 ± 2.69	23.7 ± 7.23	19.1 ± 3.52
IVRT (ms)	14.3 ± 2.65	13.6 ± 1.75	15.8 ± 2.64	16.0 ± 1.97	17.1 ± 3.14	14.7 ± 1.30

Echocardiographic measurements of left ventricular dimensions in para-sternal long axis (PLAX) B-mode or M-mode. HR, heart rate; SV, stroke volume, CO, cardiac output; EDV, end-diastolic volume; ESV, end-systolic volume; AWd/s, anterior wall thickness in diastole/systole; LVIDd,/s left ventricular inner diameter in diastole/systole; PWd/s, posterior wall thickness in diastole/systole; EF, ejection fraction; FS, fractional shortening; E/A, ratio of E and A peak wave velocity; Decel time, deceleration time of E wave from peak to projected baseline; IVRT, isovolumetric relaxation time .Statistics used One-way repeated ANOVA with

Tukey's *post-hoc* test.*$P<0.05$ between RD and O304 at same timepoint. #$P<0.05$ compared to baseline. ¤$P<0.05$ compared to scan 2. Data are mean±SD.

Supplementary table 2. Antibodies

Immunohistochemistry				
	**Antigen**	**Species**	**Supplier (cat.nr.)**	
Primary antibodies	Insulin	Guinea pig	Dako (A0564)	1:500
	Glucagon	Rabbit	Euro Diagnostica (B31-1)	1:1000
	Somatostatin	Rabbit	Abcam (ab111912)	1:5000
	Pancreatic Polypeptide	Guinea pig	Linco (4041-01)	1:500
	Glut2	Rabbit	Produced in house (Goulley et al., Cell Metabolism 5:207–219, 2007)	1:400
	Ipf1/Pdx1	Rabbit	Produced in house (Ohlsson et al., EMBO J 12:4251–4259. 1993)	1:800
	Nkx6-1	Rabbit	Produced in house (Öström et al., PloSONE 3:e2841, 2008)	1:1000
	MafA	Rabbit	Nordic Biosite (IHC-00352)	1:500
	Raldh3	Rabbit	Novus Biologicals (NBP2-15339)	1:1000
secondary antibodies	Alexa Fluor® 594 AffiniPure Donkey Anti-Rabbit IgG (H+L)	Donkey	Jackson ImmunoResearch. (711-585-152)	1:500
	Alexa Fluor® 488 AffiniPure Donkey Anti-Guinea Pig IgG (H+L)	Donkey	Jackson ImmunoResearch. (706-545-148)	1:1000
**Western Blot**				
	**Antigen**	**Species**	**Supplier (cat.nr.)**	**Dilution and blocking**
Primary antibodies	P-Acc (Ser79)	Rabbit	Cell Signaling (#3661)	1:1000 in 5% BSA
	Acc	Rabbit	Cell Signaling (#3662)	1:1000 in 5% BSA
	P-Raptor (Ser792)	Rabbit	Cell Signaling (#2083)	1:1000 in 5% BSA
	Raptor	Rabbit	Cell Signaling (#2280)	1:1000 in 5% BSA
	P-Ampk (Thr172)	Rabbit	Cell Signaling (#2535)	1:2000 in 5% BSA
	Ampk	Rabbit	Cell Signaling (#2532)	1:2000 in 5% BSA
	Txnip	Rabbit	Abcam (ab188865)	1:1000 in 5% non-fat dry milk
	Beta-actin	Rabbit	Cell Signaling (#4967)	1:1000 in 5% BSA
	P-S6 (Ser240/244)	Rabbit	Cell Signaling (#5364)	1:50.000 in 5% BSA
	S6	Rabbit	Cell Signaling (#2217)	1:20.000 in 5% BSA
secondary antibodies	HRP-anti-Rabbit	Goat	Jackson ImmunoResearch. (111-035-003)	1:10.000 in 2,5% BSA or 5% milk

Supplementary table 3. Human islets donors

	Age (years)	Gender	BMI (kg/m2)	HbA1c (mmol/ml)	Stimulatory index (SI)
Donor #1	52	M	26.9	37	Not known
Donor #2	56	F	24.6	40	7.9
Donor #3	55	M	22.4	36	10
Donor #4	43	M	18.9	33	Not known
Donor #5	65	M	29.4	43	Not known

Supplementary table 4. Primers

Entrez_ gene ID	Symbol	Alias	Forward primer	Reverse primer
56847	*Aldh1a3*	*RaldH3*	CCGAGCGATCCTGGCTACTC	GAAGGAATGGCTTGCCGGTG
12869	*Cox8b*	*Cox8b*	TCTGCCAAGCCAGCCAAAAC	AGCCTGCTCCACGGCG
108687	*Edem2*	*Edem2*	ACTTGGGAGAGACGCTGTGG	GGAGGTCCTTGATCGTGGCA
67397	*Erp29*	*Pdi*	AGCTCTTGGTGGCAGAGGTG	CATGTTCAGCTTGTCGCCAT
15277	*Hk2*	*Hk2*	GGATGACCTGCGCACAGTG	ATCAGGATGTTGCGCACAATC
3309	*Hspa5*	*Bip*	CGAGGAGGAGGACAAGAAGG	CACCTTGAACGGCAAGAACT
16333, 16334	*Ins1,2*	*Ins1,2*	CCACCCAGGCTTTTGTCAA	TCCCCACACACCAGGTAGA
378435	*Mafa*	*MafA*	GCTGCTGCGGCCTATGAG	TGGCCGGCACCCATGT
18096	*Nkx6-1*	*Nkx6.1*	GCACGCTTGGCCTATTCTCT	TCTCGGCTGCGTGCTTCT
18597	*Pdha1*	*Pdha1*	TTGCAGGTCTGGTAAGGGGC	TGGTAGCGGTAAGTCTGGAGC
27273	*Pdk4*	*Pdk4*	ACTCCACTGCTCCAACACCTG	ACCAAAACCAGCCAAAGGGG
18609	*Pdx1*	*Ipf1*	GACACATCAAAATCTGGTTCCAAA	GGTCCCGCTACTACGTTTCTTATC
18746	*Pkm*	*Pkm*	TTTGTACCATTGGGCCTGCT	TTCAGCCGAGCCACATTCAT
19017	*Ppargc1a*	*PGC1a*	GAGAAGCGGGAGTCTGAAAGG	TCACACGGCGCTCTTCAAT
66945	*Sdha*	*Sdha*	CATCCCAGTCCTCCCCACTG	CATGCTTCAGCACCTGTCCC
20525	*Slc2a1*	*Glut1*	TTATTGCCCAGGTGTTTGGC	ACAAGTCTGCATTGCCCATG
20526	*Slc2a2*	*Glut2*	TCCTCGTGGCGCTGATG	CTGGTTGAATAGTAAAATATCCCATTGAT
20528	*Slc2a4*	*Glut4*	TCATTGTCGGCATGGGTTT	GGCAAATAGAAGGAAGACGTAAGG
21374	*Tbp*	*Tbp*	GAATTGTACCGCAGCTTCAAAA	AGTGCAATGGTCTTTAGGTCAAGTT
56843	*Trpm5*	*Trpm5*	TGCTCAAGCAGGTCTTCAGG	TCTTCTGGTCCAAGGGGTCA
56338	*Txnip*	*Txnip*	TCTTTATGTACGCCCCTGAGTTC	TTAAGGACGCACGGATCCA
83428	*Ucn3*	*Ucn3*	CCACTTACAGGGAGCGATGC	CAGCAGGAAGTAGGTGGGCA
22631	*Ywhaz*	*Ywhaz*	CTGCGTGACATCTGCAACGA	GGTTGCGAAGCATTGGGGAT
19951	*Rpl32*	*Rpl32*	TGCTGCTGATGTGCAACAAATCT	TGTGAGCAATCTCAGCACAGT
