# Supplementary material for: O304 ameliorates hyperglycemia in mice by dually promoting muscle glucose effectiveness and preserving β-cell function
Source: Commun Biol. 2023 Aug 25;6:877. doi: 10.1038/s42003-023-05255-6 (PMC10457357; doi:10.1038/s42003-023-05255-6)
Supplement: Supplementary file 2 — Description of Additional Supplementary Files [file 42003_2023_5255_MOESM2_ESM.pdf]

Description of Additional Supplementary Files

File name: Supplementary Data 1
Description: The source data behind the curves, graphs, MA plots, and heatmaps in figures 1-7.
